# A Novel TGFβ Modulator that Uncouples R-Smad/I-Smad-Mediated Negative Feedback from R-Smad/Ligand-Driven Positive Feedback

**DOI:** 10.1371/journal.pbio.1002051

**Published:** 2015-02-09

**Authors:** Wenchao Gu, Rui Monteiro, Jie Zuo, Filipa Costa Simões, Andrea Martella, Charlotte Andrieu-Soler, Frank Grosveld, Tatjana Sauka-Spengler, Roger Patient

**Affiliations:** 1 Weatherall Institute of Molecular Medicine, University of Oxford, John Radcliffe Hospital, Oxford, United Kingdom; 2 BHF Centre of Research Excellence, Oxford, United Kingdom; 3 Department of Physiology, Anatomy and Genetics, University of Oxford, Oxford, United Kingdom; 4 Department of Cell Biology, Erasmus Medical Centre, Rotterdam, The Netherlands; 5 INSERM U872, Université René Descartes Sorbonne Paris Cité, Team 17, Centre de Recherche des Cordeliers, Paris, France; London Research Institute, UNITED KINGDOM

## Abstract

As some of the most widely utilised intercellular signalling molecules, transforming growth factor β (TGFβ) superfamily members play critical roles in normal development and become disrupted in human disease. Establishing appropriate levels of TGFβ signalling involves positive and negative feedback, which are coupled and driven by the same signal transduction components (R-Smad transcription factor complexes), but whether and how the regulation of the two can be distinguished are unknown. Genome-wide comparison of published ChIP-seq datasets suggests that LIM domain binding proteins (Ldbs) co-localise with R-Smads at a substantial subset of R-Smad target genes including the locus of inhibitory *Smad7* (I-Smad7), which mediates negative feedback for TGFβ signalling. We present evidence suggesting that zebrafish Ldb2a binds and directly activates the *I-Smad7* gene, whereas it binds and represses the ligand gene, *Squint (Sqt)*, which drives positive feedback. Thus, the fine tuning of TGFβ signalling derives from positive and negative control by Ldb2a. Expression of *ldb2a* is itself activated by TGFβ signals, suggesting potential feed-forward loops that might delay the negative input of Ldb2a to the positive feedback, as well as the positive input of Ldb2a to the negative feedback. In this way, precise gene expression control by Ldb2a enables an initial build-up of signalling via a fully active positive feedback in the absence of buffering by the negative feedback. In Ldb2a-deficient zebrafish embryos, homeostasis of TGFβ signalling is perturbed and signalling is stably enhanced, giving rise to excess mesoderm and endoderm, an effect that can be rescued by reducing signalling by the TGFβ family members, Nodal and BMP. Thus, Ldb2a is critical to the homeostatic control of TGFβ signalling and thereby embryonic patterning.

## Abstract 2

A LIM domain binding protein mediates both positive and negative feedback to fine-tune the regulation of transcription in response to transforming growth factor β signalling during embryonic development.

## Author Summary

Cells depend on signals from their microenvironment to carry out their normal functions and coordinate responses. Once initiated, such signals often self-amplify via positive feedback to reach a sufficient level, when negative feedback can then be employed to dampen excess signalling. These feedback loops dynamically add or remove signalling components to maintain homeostasis. Their activation is often driven by the same signal transduction components, making it difficult to understand how signalling builds up in the first place. Here we find that the transcription co-factor Ldb2a enables differential response dynamics of negative and positive feedback upon the induction of TGFβ signalling. We show that Ldb2a directly activates expression of a TGFβ inhibitor that mediates negative feedback, while also repressing expression of TGFβ ligands that drive positive feedback. Moreover, expression of *Ldb2a* is itself activated by TGFβ signals. Thus, when Ldb2a levels are initially low, TGFβ signalling can self-amply and build up signal via positive feedback without being countered by negative feedback. We show that this regulatory mechanism is active in developing zebrafish embryos, where a loss of Ldb2a results in the over production of mesodermal and endodermal tissue types as a consequence of elevated TGFβ family signalling.

## Introduction

In vertebrates, the transforming growth factor β (TGFβ) superfamily comprises a large number of ligands, including TGFβs, Nodal, Activin, and bone morphogenetic proteins (BMPs), each of which can direct lineage-specific transcriptional responses that regulate biological processes as diverse as cell proliferation, differentiation, apoptosis, and severe diseases caused by their mis-regulation [1]. In response to extracellular ligand binding, trans-membrane receptors phosphorylate receptor-activated Smads (R-Smads) in the cytoplasm. Different ligand-stimulated pathways converge and signal through two main R-Smad pathways, with Nodal/TGFβ/Activin mediated by R-Smad2/3 and BMP by R-Smad1/5/8 [2]. Activated R-Smads interact with the common partner Smad4 (Co-Smad4) to carry the signals into the nucleus, where the Smad complexes associate with additional transcription factors (TFs) and co-factors, as well as co-activators or co-repressors, to regulate downstream target genes [3].

The level of TGFβ signalling is established by homeostatic regulation, which dynamically adds or removes signalling components to maintain a sufficient and constant level of activity. For example, TGFβ signals activate expression of their own ligands [4–9]. After secretion from the cell, these ligands bind transmembrane TGFβ receptors, implementing positive feedback to self-amplify and sustain signals at a sufficient level and to propagate the signals into neighbouring cells. The inhibitors of TGFβ signalling, such as Leftys and inhibitory Smad6 and Smad7 (I-Smad6/7), can also be induced by TGFβ family signals, thereby generating negative feedback to dampen excess signalling [8–12]. These positive and negative feedbacks are coupled, as the TGFβ-responsive induction of both is by direct binding of R-Smads and Co-Smad4 to ligand or inhibitor genes [2,6,8,9,13–17]. Activation of TGFβ family signalling pathways results in rapid recruitment of transcriptional co-activators to ligand and *I-Smad* genes, leading to their up-regulation *in vivo* [8,9]. In zebrafish, the expression of Nodal ligand genes and *Smad7* can be induced by *R-Smad3* expression [12]. It has been demonstrated that coupled positive and negative feedback confers flexibility on signal switches and enables precise modulation of signal responses [18–20]. However, whether and how the activation of negative and positive feedbacks can be uncoupled is not known.

LIM domain binding proteins (Ldbs) are multi-functional non-DNA binding adaptor proteins that assemble TF complexes on target genes [21–25]. Components of such Ldb complexes, Lmo4 and Gata1/2 for example, have been shown to recruit R-Smad complexes onto TGFβ target genes [9,26,27]. By comparing published chromatin immunoprecipitation (ChIP)-seq datasets of genome-wide protein-DNA binding profiles for R-Smad1/3 and Ldb1 [8,9,21], we have obtained evidence that Ldb1 co-localises with R-Smad1/3 at a substantial subset of R-Smad target sites across the genome, suggesting that Ldb1 might function together with R-Smads to implement transcriptional responses to TGFβ family signalling. In vertebrates, a paralogue, Ldb2, shares a high percentage of amino acid sequence identity and structural similarity with Ldb1 [28], but its functions are largely unknown. In this study, we present *in vivo* functional and phenotypic data showing that Ldb2 regulates Nodal/BMP signalling and is required for early embryogenesis. Furthermore, we identify *I-Smad7* and a Nodal ligand, *Sqt*, as direct target genes activated or suppressed respectively by Ldb2a, and show that the fine tuning of TGFβ family signalling requires both positive and negative control by Ldb2a complexes.

## Results

### Ldbs and R-Smads Co-localise at a Subset of TGFβ Target Genes

**Fig 1:**
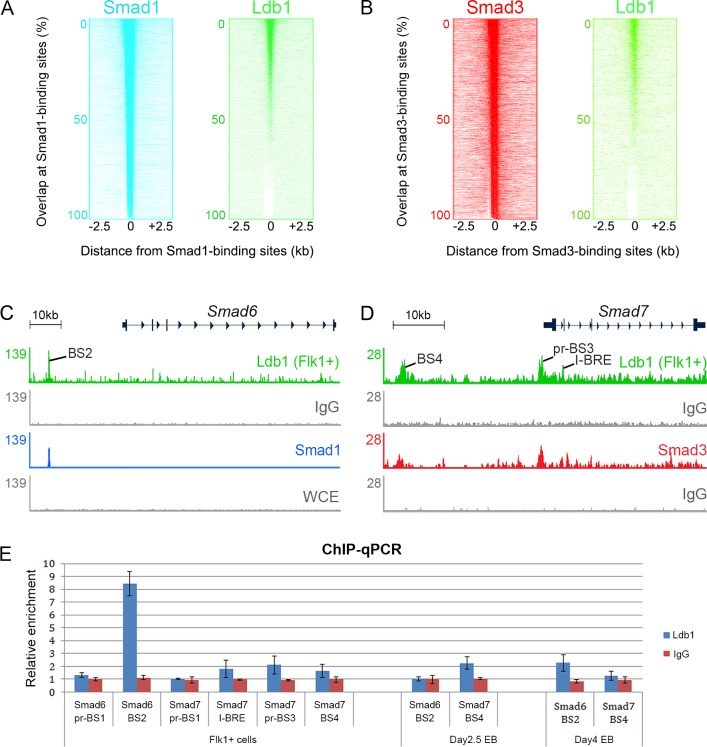
Ldb1, R-Smad1, and R-Smad3 share a substantial subset of target genes across the genome. Genome-wide comparison of different ChIP-seq datasets shows that Ldb1, R-Smad1, and R-Smad3 co-localise at a substantial subset of R-Smad binding sites. (A) For each R-Smad1 binding site (*y*-axis), the relative locations of sites bound by R-Smad1 itself, as the positive control (blue), and Ldb1 (green) are displayed within a 5-kb window centred on the R-Smad1 binding site (position 0). High intensity at position 0 indicates co-occupancy. (B) For each R-Smad3 binding site (*y*-axis), the relative locations of sites bound by R-Smad3 itself (red) and Ldb1 (green) are displayed. (C) Ldb1 and R-Smad1 co-localise at the *I-Smad6* locus. (D) Ldb1 and R-Smad3 co-occupy the *I-Smad7* locus. ChIP-seq datasets analysed in (A–D) were obtained in different cell types, with Ldb1 in murine Flk1+ cells, and R-Smad1 in murine G1ER cells. R-Smad3 ChIP-seq was performed in murine pro-B cells. (E) Ldb1 binding was enriched at the *Smad6* BS2 region (see C) and the *Smad7* promoter (pr-BS3, see D) regions in murine day 4 EB-derived Flk1+ cells. In day 2.5 EBs, Ldb1 was enriched at *Smad7* and at *Smad6* in day 4 EBs. Shown as the negative control, Ldb1 binding was not enriched at *Smad6* pr-BS1, *Smad7* pr-BS1, *Smad7* I-BRE, and *Smad7* BS4 in Flk1+ cells, neither at Smad6 BS2 in day 2.5 EBs, nor at Smad7 BS4 in day 4 EBs. Error bars (standard deviation [SD]) are based on three biological replicates, each with three technical replicates.

We compared published ChIP-seq datasets of Ldb1, the BMP effector, R-Smad1, and the Nodal/Activin/TGFβ effector, R-Smad3 [8,9,21,29]. We found that the binding of Ldb1 overlaps R-Smad1 or R-Smad3 binding at a substantial subset of R-Smad targets across the genome (Fig. 1A and 1B), including at the known TGFβ target genes, *I-Smad6* and *I-Smad7* (Fig. 1C and 1D). Ldb1 binding at these loci was validated in murine cells by ChIP-quantitative PCR (qPCR) (Fig. 1E). The ChIP-seq of Ldb1 had been performed in murine bone marrow cells or day 4 embryoid body (EB)-derived Flk1+ haemato-endothelial precursor cells [21,29], whereas the ChIP-seq of R-Smad1 and R-Smad3 had been carried out in murine G1ER erythroid progenitor cells and murine pro-B cells, respectively [8,9]. Nevertheless, the widespread co-localisation of Ldb1 and R-Smads, albeit in different cell types, suggests the potential for functional cooperation between these factors.

Ldb1 does not bind DNA directly but has been shown to assemble complexes containing Scl (Tal1) and Gata1/2 on DNA via motifs including Ebox, GATA, and Ets [23,24,29]. Genome-wide comparison of ChIP-seq datasets suggests that Scl and Gata1/2 co-occupy a substantial subset of Ldb1-binding sites with R-Smad1 or R-Smad3 (S1 Fig.). Indeed, the most enriched motifs identified in genomic sequences bound by R-Smads also include GATA, Ebox and Ets [8,9]. Taken together, these observations identify Ldb proteins as potential modulators of TGFβ superfamily signalling, possibly by associating with R-Smads to regulate TGFβ targets.

**Fig 2:**
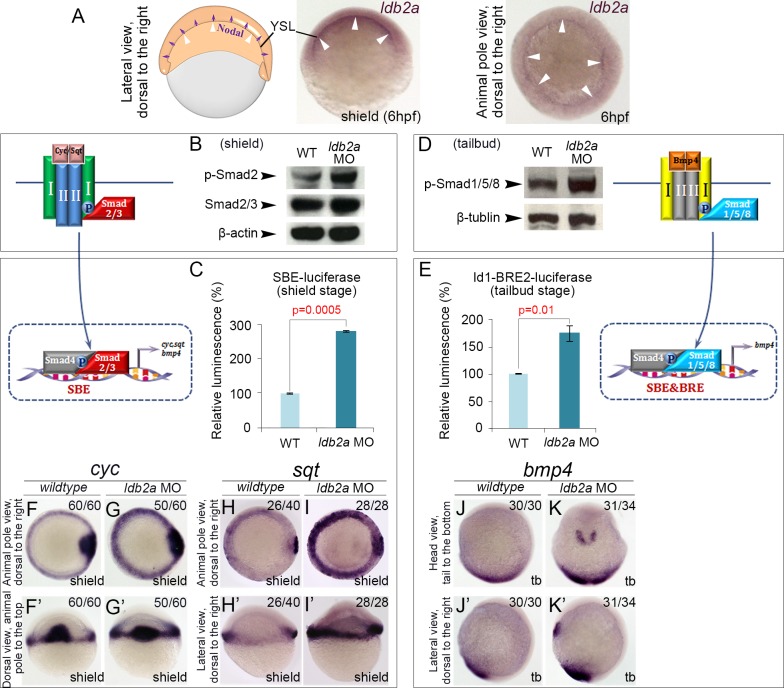
Knockdown of *ldb2a* enhances Nodal/BMP signal transduction in zebrafish embryos. (A) At the onset of gastrulation, *ldb2a* expression is more noticeable in the yolk syncytial layer (YSL) (white arrowheads), which is an important source of Nodal signals (purple arrows). (B) At the shield stage, western blot showed increased phosphorylation of R-Smad2 in *ldb2a* morphants, while the level of overall Smad2/3 remained the same. β-actin was used as a loading control. (C) The activity of a TGFβ reporter SBE-luciferase was up-regulated in shield stage *ldb2a* morphants. (D) Phosphorylation of R-Smad1/5/8 was up-regulated in *ldb2a* morphants at the tailbud stage. β-tubulin was the loading control. (E) The activity of a BMP reporter Id1-BRE2-luciferase was increased in tailbud stage *ldb2a* morphants. (C and E) each displays a single representative experiment of three biological replicates, with the error bars corresponding to two technical replicates. For each biological replicate, 50 embryos were lysed and analysed. (F–I’) Expression of Nodal ligands, *cyc* and *sqt*, was increased in shield stage *ldb2a* morphants. (J–K’) Anterior and posterior expression of *bmp4* was up-regulated at the tailbud stage. (F–G’): seven independent experiments, with the total number of analysed embryos shown on the top-right corner of each panel; (H–K’): two independent experiments. The *wildtype* control refers to uninjected embryos that are stage matched.

To analyse the role of Ldbs in TGFβ signalling *in vivo*, we first monitored their expression during early embryonic development when TGFβ family members are known to be critical. Throughout early zebrafish development, *ldb2a* shows greater specificity than the ubiquitous *ldb1a*, *ldb1b*, or *ldb2b* (S2 Fig. and data retrieved from the Zebrafish Information Network (ZFIN) [30]). At 15 hours post fertilisation (hpf), *ldb2a* is present in the notochord and the lateral mesoderm, which gives rise to haematopoietic, endothelial, and pronephric derivatives (S2A Fig.). At 26 hpf, *ldb2a* expression continues in and around the blood vessels (S2B Fig.). Maternal/zygotic *ldb2a* is expressed ubiquitously throughout cleavage and blastula stage (0–4.7 hpf) embryos (S2C–S2F Fig.), but immediately before and during gastrulation (4.7–10 hpf), *ldb2a* becomes more specific in the yolk syncytial layer (YSL) (Figs. 2A and S2F, white arrowheads), an important source of Nodal signalling crucial for the specification of gastrula germ layers. This suggests a possible role for Ldb2a in signalling by this TGFβ superfamily member, we therefore initially focussed our studies on the function of Ldb2a in Nodal signalling during gastrula embryonic development.

### Knockdown of *ldb2a* Enhances Nodal/BMP Signal Transduction and Ligand-Mediated Positive Feedback

To determine if Ldb2a functions in Nodal signal transduction (illustrated in Fig. 2B–2E), we knocked it down using two antisense morpholinos (MOs), a splice MO targeting the boundary of intron3 and exon4, and a MO targeting the ATG site (S3A–S3E Fig.). Both MOs cause similar defects (S3F–S3K Fig.), and co-injection of *ldb2a* mRNA with the splice MO was able to rescue *ldb2a* morphant phenotypes (S3L–S3T Fig.). Moreover, we injected NLS-Cas9 protein together with a small guide RNA targeting the ATG of *ldb2a*, and observed that a significant proportion of resultant mosaic F_0_ mutants phenocopy the morphants (S3U–S3W Fig.). Altogether, these data confirm the specificity of the *ldb2a* MOs. Upon *ldb2a* knockdown, we saw an increase in the level of the phosphorylated Nodal effector, p-Smad2, by the shield stage (6 hpf), while the level of total Smad2/3 was comparable to the wild-type control (Fig. 2B). We also observed up-regulated activity of a TGFβ reporter (SBE-luciferase [31]) (Fig. 2C). Thus, *ldb2a* knockdown up-regulates Nodal signalling, suggesting that Ldb2a normally acts to suppress Nodal signalling.

Another TGFβ superfamily member, BMP, plays critical roles during gastrulation and signals through R-Smad1, which also co-occupies the genome with Ldb1 (Figs. 1A and S1A). We therefore examined the BMP signal transduction pathway in *ldb2a* morphants. The activity was unaffected at the shield stage (S4A and S4B Fig.) but significantly increased by the end of gastrulation (the tailbud stage, 10 hpf), as shown by the level of p-Smad1/5/8 and the activity of a BMP-specific reporter (Id1-BRE2-luciferase [32]) (Fig. 2D and 2E). Thus, *ldb2a* loss-of-function promotes BMP signal transduction, suggesting that Ldb2a normally acts to suppress BMP signalling.

The consequences of the excessive Nodal signalling in *ldb2a* morphants included up-regulation of the Nodal-induced genes, *cyclops (cyc)* and *squint* (*sqt)* (Fig. 2F–2I’). Expression of *bmp4* was also increased by the tailbud stage (Fig. 2J–2K’) and remained up-regulated during somitogenesis (S4C and S4D Fig.). These genes code for ligands that implement positive feedback to sustain and propagate signalling. Taken together, *ldb2a* knockdown enhances expression of Nodal and BMP ligands, suggesting a negative effect of Ldb2a on positive feedback for Nodal and BMP signalling.

### Embryonic Patterning Depends on Ldb2a Modulation of Nodal and BMP Signalling

**Fig 3:**
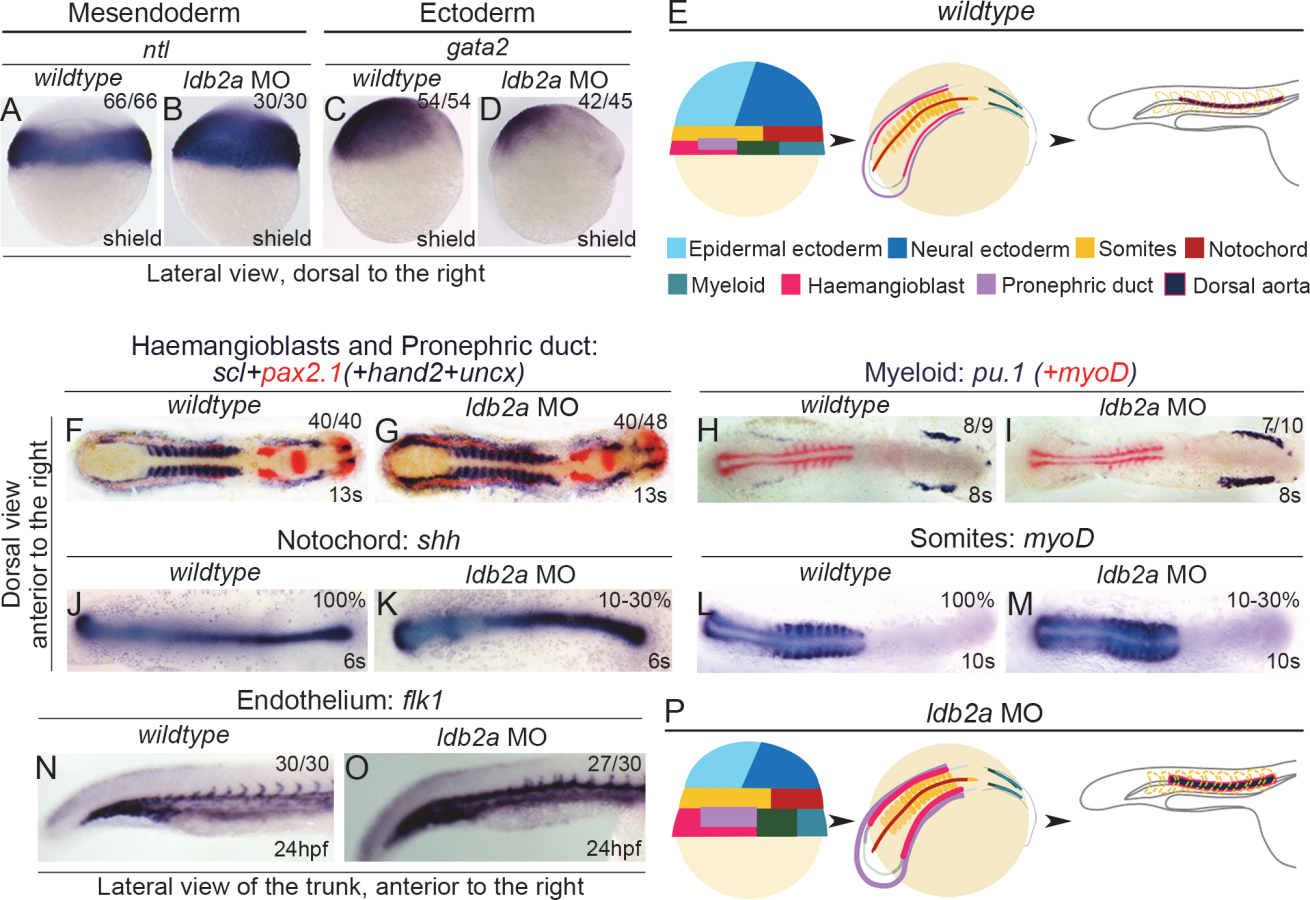
Ldb2a knockdown promotes the specification of mesendoderm and mesoderm. (A–B) In *ldb2a* morphants, *ntl* expression in the mesendoderm/mesoderm was expanded into the ectoderm. (C–D) The ectoderm was restricted (decreased *gata2* expression). (E) Gastrula ventral or dorsal mesendoderm develops into haemangioblast/pronephric or myeloid populations respectively, during somitogenesis, while the mesoderm becomes somites and notochord. (F–I) During somitogenesis, expression of *scl*, *pax2*.*1*, *hand2*, *and pu*.*1* in the lateral mesoderm was up-regulated in *ldb2a* morphants. Embryos were co-stained with *uncx* or *myoD* to define the stage. (J–M) Other mesendoderm and mesoderm derivatives were also increased including somite (*myoD*) and notochord (*shh*), although only in a proportion of most affected morphants. (N–O) At 24 hpf, *ldb2a* knockdown caused increased expression of an endothelial gene, *flk1*, in the vessels (lateral mesoderm-derived). (P) As a summary, the loss of *ldb2a* expression induces mesodermal and endodermal while restricting ectodermal fates, especially in the ventro-lateral and posterior regions, and this fate change is stable. (A–D): three independent experiments, with the total number of analysed embryos shown in each panel; (F–G): five independent experiments; (H–I): one experiment; (J–O): three independent experiments. The *wildtype* control refers to uninjected embryos that are stage matched.

In addition to the expression of ligands, readout of Nodal signalling also includes expression of various germ layer genes, as Nodal induces the mesendoderm while restricting the ectoderm [33–35]. Consistent with the excessive Nodal signalling observed in *ldb2a* morphants, expression of *ntl*, a mesendoderm marker, was expanded towards the presumptive ectoderm (Fig. 3A and 3B), while expression of *gata2*, a non-neural ectoderm marker, and *otx2*, a neural ectoderm marker, was reduced (Figs. 3C, 3D, S5A–S5B’). In addition, another Nodal target, *mixer/bon*, expressed in the mesendoderm at the onset of gastrulation and becoming restricted to the endoderm during late gastrulation [36,37], and critical for proper endoderm specification in a Nodal-dependent manner [38], displayed increased expression in *ldb2a* morphants at the shield and 80% epiboly stages, suggesting a critical role for Ldb2a in the specification of endoderm (S5C–S5F’ Fig.). Taken together, these data suggest that some of the ectoderm is converted to mesoderm and endoderm in *ldb2a* morphants, consistent with the excessive Nodal signalling observed in these embryos.

To monitor the stability of the patterning effects of Ldb2a via Nodal, we examined genes expressed in mesendoderm-derived tissues of *ldb2a* morphants at later stages. At the 13-somite stage (∼15 hpf), markers of the mesendoderm-derived lateral mesoderm, such as a lateral mesodermal gene, *hand2*, a pronephric duct gene, *pax2*.*1*, and a haemangioblast gene, *scl*, displayed up-regulated expression in *ldb2a* morphants (Fig. 3F and 3G). We also observed up-regulation of other lateral mesodermal genes, including the haemangioblast genes *lmo2*, *gata2*, and *fli1*, erythroid genes *gata1* and *draculin*, a myeloid gene *pu*.*1*, and the pronephric duct genes *pax8* and *lim1* (Figs. 3H, 3I, and S6A–S6N). To quantify expression of genes in the lateral mesoderm, we performed quantitative real-time PCR (qPCR) analyses and observed an increased level of *fli1* RNA in *ldb2a* morphants at the 12-somite stage (S6O Fig.). In addition, Tg(gata1a:GFP)^la781^ zebrafish embryos injected with the *ldb2a* MO showed a clear up-regulation of GFP expression (S6P and S6Q Fig.), indicating an increase in the protein level of Gata1, but also in the number of Gata1 positive cells.

Consistent with the unchanged BMP activity at the beginning of gastrulation, dorsoventral patterning of *ldb2a* morphants remained balanced, shown by increased expression of both a ventral mesendoderm marker, *eve1*, and a dorsal mesendoderm marker, *gsc* (S5G–S5J’ Fig.). However, the activity of BMP signalling and expression of *bmp4* became up-regulated in *ldb2a* morphants during late gastrulation (Fig. 2D, 2E, 2J, and 2K’), when high level BMP continues to specify ventral and posterior mesodermal tissues. After gastrulation, we indeed observed increased expression of genes marking the lateral mesoderm, derived from the ventro-posterior mesoderm (Figs. 3F–3I and S6).

To further investigate the effects of Ldb2a activity via a combination of Nodal and BMP after gastrulation, we examined expression of paraxial and dorsal mesodermal genes in *ldb2a* morphants. They were indeed up-regulated (by excessive Nodal) but less severely compared to the ventrally expressed genes (influenced by both Nodal and BMP), as shown by increased expression of *shh* (notochord) and *myoD* (somite) in the 10%–30% most affected *ldb2a* morphants (Fig. 3J–3M). Furthermore, the effect of *ldb2a* knockdown in the ventro-lateral mesendoderm-derived tissues remained evident until 24 hpf, when we observed up-regulated expression of *flk1*, *tie1*, *dll4*, and *deltaC* in endothelial cells of *ldb2a* morphants (Figs. 3N, 3O, and S7A–S7F). Taken together, our findings indicate that *ldb2a* loss-of-function induces mesodermal and endodermal while restricting ectodermal fates, especially in the ventro-lateral regions, and that this fate change is stable (Fig. 3P).

**Fig 4:**
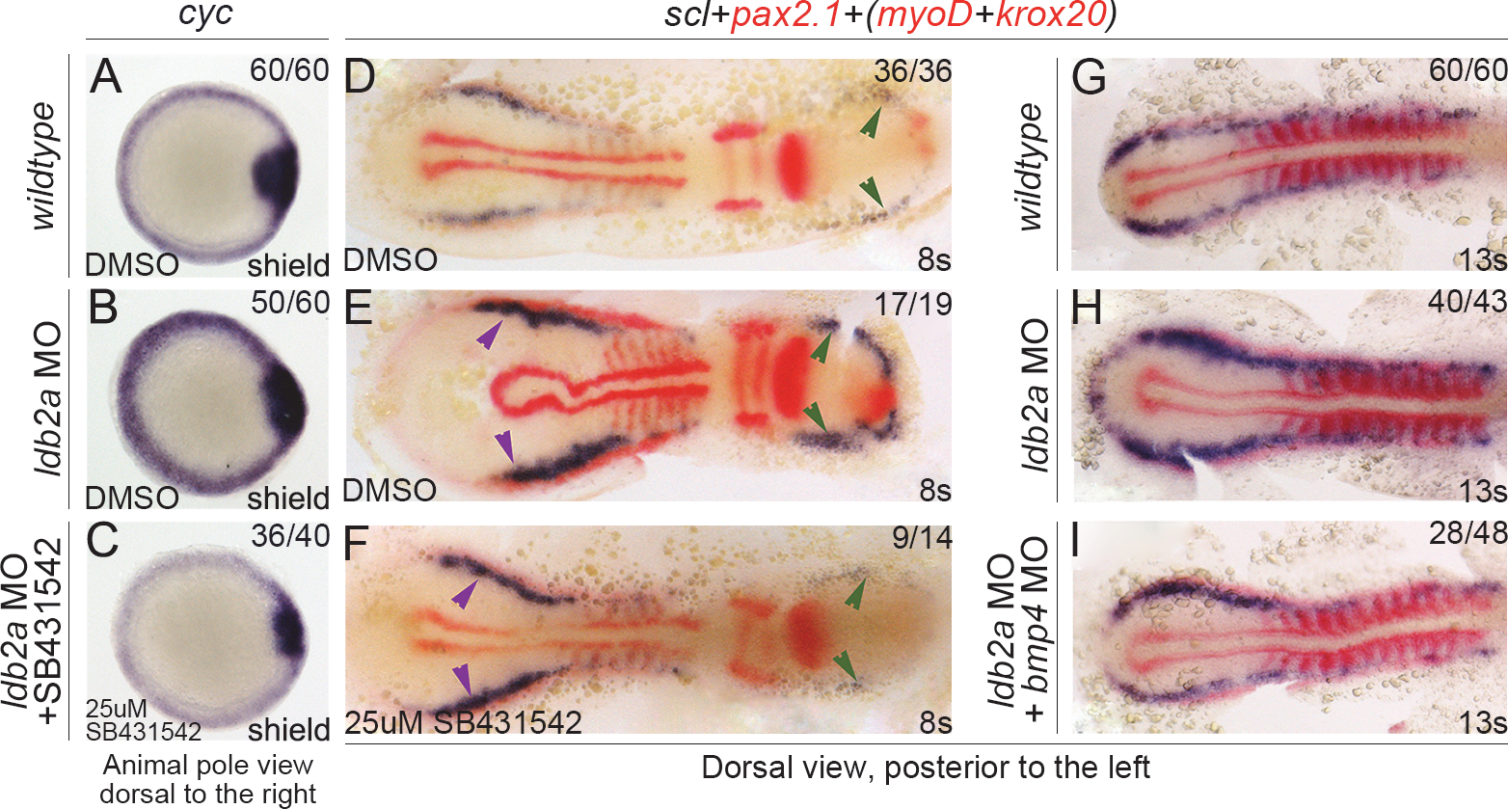
The roles of Ldb2a in the specification of mesendoderm and derivatives depend on Nodal and BMP signalling. (A–C) SB431542 rescued the increased expression of *cyc* caused by *ldb2a* knockdown. (D–F) SB431542 also partially rescued the increase in expression of lateral mesodermal genes, *scl* and *pax2*.*1*, in *ldb2a* morphants (green and purple arrowheads). The *wildtype* control refers to uninjected embryos that are stage matched and treated with equal volume of DMSO. (G–I) Co-injection with a *bmp4* MO partially rescued the increased *scl* expression in *ldb2a* morphants. Embryos co-stained with *myoD* and *krox20* to define the stage and territory. (A–C): three independent experiments (the morphant phenotype of *cyc* expression was observed from seven independent experiments, whereas the rescue experiments were repeated three times); (D–F): two independent experiments; (G–I): three independent experiments. The *wildtype* control refers to uninjected embryos that are stage matched.

To confirm that the ectopic mesendoderm formation in *ldb2a* morphants is due to the up-regulation of Nodal and BMP signalling, we tried to reverse the effects by reducing these signals. When treated with an Alk4/5/7 (Nodal/Activin/TGFβ receptors) inhibitor, SB431542, *ldb2a* morphants were rescued with respect to ectopic expression of *cyc* (Fig. 4A–4C) and of *scl* and *pax2*.*1* (Fig. 4D–4F). Moreover, *bmp4* knockdown by MO injection also rescued the increased expression of *scl* and *pax2*.*1* in *ldb2a* morphants (Fig. 4G–4I). These observations suggest that Ldb2a functions through Nodal signalling to restrict the specification of mesendoderm and through BMP signalling to restrict the specification of ventro-lateral mesendoderm.

### Ldb2a Is Required for I-Smad7-Mediated Negative Feedback

**Fig 5:**
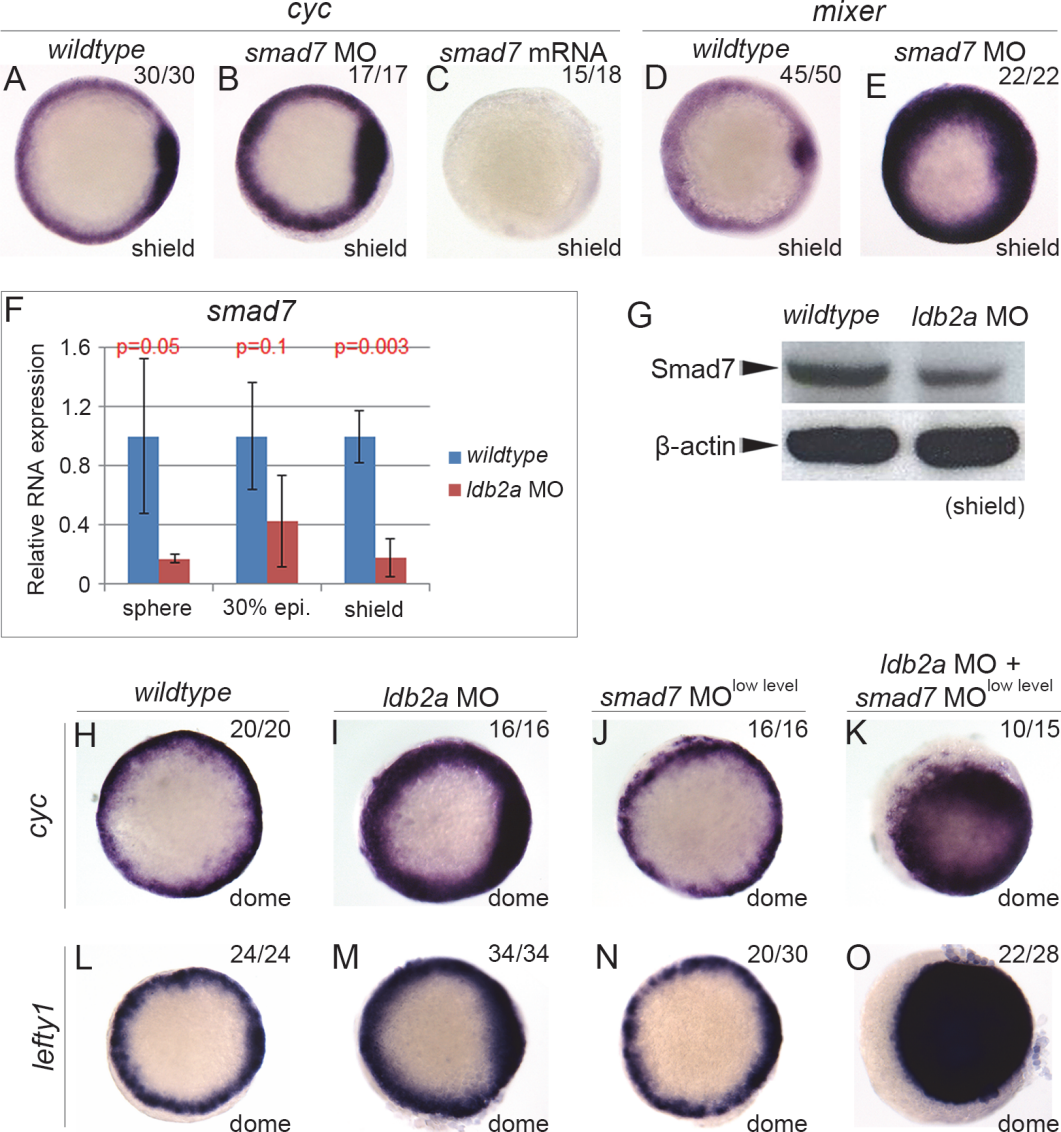
Ldb2a and Smad7 function in the same pathway. Knockdown of *smad7* increased expression of Nodal target genes, *cyc* (A–B) and *mixer* (D–E), whereas overexpression of *smad7* mRNA abolished *cyc* expression (C). In (A–E), one experiment was performed, with the total number of analysed embryos indicated in each panel. These are mainly confirming results published already [12,39]. (F) *Smad7* mRNA level decreased in *ldb2a* morphants from the sphere stage, shown by RT-qPCR. Error bars are drawn from three independent experiments, each with three technical replicates. (G) Western blot showed decreased protein levels of Smad7 in shield stage *ldb2a* morphants. β-actin was the loading control. (H–O) Co-injection of *ldb2a* MO with low-level *smad7* MO that did not cause any defect on its own resulted in ectopic expression of Nodal targets, *cyc* and *lefty1*, in the ectoderm, suggesting a synergistic effect between *ldb2a* and *smad7* MOs. Experiments were repeated twice, with the total number of analysed embryos indicated in each panel. The *wildtype* control refers to uninjected embryos that are stage matched.

Under normal circumstances, once Nodal signalling is up-regulated, negative feedback dampens excess signalling. However, the fact that a stable Nodal-dependent effect of *ldb2a* knockdown was seen suggests that negative feedback might not be fully active. Such feedbacks for both Nodal and BMP can be mediated by their common inhibitor, I-Smad7 [10–12,39]. Smad7 antagonises Nodal and BMP signal transduction via multiple mechanisms, dampening the phosphorylation of R-Smads, the formation of R-Smad/Co-Smad4 complexes, or the binding of R-Smad/Co-Smad4 to DNA [40–45]. By causing disruption of these mechanisms, altered Smad7 levels can eventually lead to changes in expression of Nodal targets, including ligand and mesendodermal genes. We first confirmed the role of Smad7 as a Nodal inhibitor in zebrafish embryos, showing that *cyc* expression was increased by *smad7* MO knockdown (S8 Fig.) [12], but decreased by *smad7* overexpression (Fig. 5A–5C). Loss-of-*smad7* also increased expression of the Nodal target, *mixer*, in the mesendoderm (Fig. 5D and 5E). We then showed that indeed Smad7-mediated negative feedback is defective in *ldb2a* morphants, as shown by decreased levels of *Smad7* mRNA and protein (Fig. 5F and 5G). Importantly, the increased *cyc* expression in *ldb2a* morphants was further up-regulated by co-injection of a level of *smad7* MO that did not give a phenotype on its own. This synergistic effect between *ldb2a* and *smad7* MOs implies that they function in the same pathway.

Leftys also mediate auto-regulatory negative feedback for Nodal signalling [4]. However, as a direct target induced by Nodal, expression of *lefty1* was increased, as opposed to decreased like *smad7*, in *ldb2a* morphants (Fig. 5L and 5M), consistent with the excessive Nodal signalling in these embryos. Moreover, Ldb2a and Smad7 are synergistic on *lefty1* expression (Fig. 5N and 5O), as seen for *mixer*. Therefore, Ldb2a is required for the negative feedback driven by Smad7 but not by Lefty1.

### Ldb2a Uncouples I-Smad7-Driven Negative Feedback from Ligand-Dependent Positive Feedback

**Fig 6:**
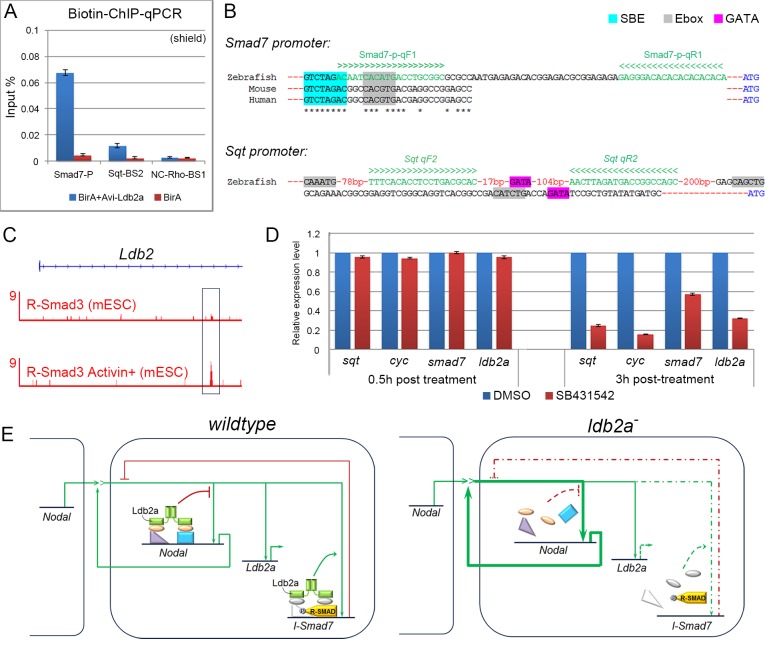
Ldb2a complexes suppress positive feedback while activating negative feedback for TGFβ family signalling. (A) ChIP-qPCR analysis detected an enrichment of Ldb2a at the promoter of *Smad7* (15-fold) and the promoter of *Sqt* (5-fold) at the shield stage. (B) SBE/Ebox motifs were found in the zebrafish *I-Smad7* promoter that is highly conserved across vertebrates, including mouse and human. GATA and Ebox motifs were found in the genomic region upstream of *Sqt*. (C) R-Smad3 is enriched at the first intron of *Ldb2* in murine ESCs, and this enrichment is increased by the addition of exogenous Activin. (D) In zebrafish, expression of *sqt*, *cyc*, *smad7*, and *ldb2a* was decreased by 100μM SB431542 treatment when examined 3 hours after the treatment from the MBT stage. Error bars are based on two independent experiments, each with three technical replicates. (E) Our findings suggest that Ldb2a associates with R-Smads to bind and activate *I-Smad7* expression in response to TGFβ family signals. Meanwhile, Ldb2a forms a repressor complex with other TFs to suppress expression of TGFβ family ligands. Thus, knockdown of *ldb2a* causes dysregulation of negative feedback, further contributing to the excessive accumulation of signalling via positive feedback that is released from the repression by Ldb2a complexes. Furthermore, Ldb2a may be involved in a coherent feed-forward loop that serves to delay the positive transcriptional response of I-Smad7 to signalling, as well as the negative response of ligand genes. Thus, negative feedback is delayed by its requirement for Ldb2a, while positive feedback is up-regulated without the repression by Ldb2a complexes. This mechanism allows signalling to quickly self-amplify until Ldb2a reaches a sufficient level to fully activate negative feedback and moderate positive feedback, thereby stabilising the level of signalling.

Upon *ldb2a* knockdown, expression of Nodal ligands and *I-smad7* was affected immediately after the mid-blastula transition (MBT) (Fig. 5F, 5I, and 5M), suggesting that the regulation of these genes by Ldb2a may be direct. Indeed, ChIP of zebrafish shield-stage embryos followed by qPCR analysis showed an enrichment of Ldb2a at the promoter of *smad7* and upstream of the *Sqt* ATG site (Fig. 6A, with primers shown in 6B). For ChIP-qPCR analysis in zebrafish, we adapted the *in vivo* biotinylation method described by de Boer and colleagues [46] for the zebrafish system. We injected low-level Avi (biotin acceptor peptide)-tagged *ldb2a* mRNA that does not cause any defect on its own (S9 Fig.), together with *NLS-BirA* (bacterial biotin ligase), in order to biotinylate Ldb2a *in vivo*; we then precipitated Biotin-Ldb2a-chromatin using streptavidin beads for subsequent analyses. We previously showed that the loss of Ldb2a exerted opposite effects on expression of different sets of genes induced by the same R-Smad pathways (i.e., down-regulation of *I-Smad7* and up-regulation of Nodal/BMP ligands). Altogether these data suggest that Ldb2a directly activates expression of *Smad7* but suppresses that of TGFβ family ligand genes, uncoupling the negative and positive feedbacks that are otherwise induced by the same R-Smad signalling.

To further explore how Ldb2a regulates expression of these genes, we mined published protein partner and DNA binding site datasets for Ldbs. Most of our current knowledge of the Ldb family is from studies of Ldb1. Since Ldb1 and Ldb2 share highly conserved protein sequence and structure, they likely function through similar mechanisms. In haematopoietic lineages, Ldb1 functions as a bridging molecule, with Lmo2/4, to assemble TF complexes that bind DNA through SBE, E-box, GATA, and Ets motifs [21,23,24]. LMO4 interacts with R-SMADs to mediate the TGFβ inputs in human epithelial cells [27]. Other components of Ldb1 complexes, such as Gata1/2, have also been shown to modulate TGFβ family signalling by assembling and recruiting Smad complexes onto TGFβ target genes [9,26]. The *Smad7* and *Sqt* genes contain conserved SBE, E-box, and GATA motifs (Fig. 6B) [47], which are known to be enriched at Smad and/or Ldb binding sites [8,9,21]. As ChIP-seq data comparison suggests that Ldb1 co-localises with R-Smad3 at the *I*-*Smad7* gene (Fig. 1D), Ldb1/2 might assemble TF complexes to recruit R-Smads to the *Smad7* locus. As previously shown, direct binding of Ldb1 at *I-Smad7* was confirmed by ChIP-qPCR in either murine EBs or Flk1+ cells (Fig. 1E), supporting our observations. Taken together, we provide evidence that Ldb2a acts together with R-Smads to bind *Smad7* at the SBE/E-box and directly activates TGFβ-induced expression of *Smad7*. On the other hand, Ldb2a suppresses *Sqt* expression, possibly via forming a repressor complex binding to the *Sqt* locus. Thus the homeostatic mechanism regulating Nodal/BMP levels of signalling requires both positive and negative control by Ldb2a complexes. Deficiency of *ldb2a* caused dysregulation of *I-smad7* expression, which subsequently disrupted the negative auto-regulating circuit, contributing to excessive activation of Nodal/BMP signalling via unrestricted positive feedback.

We conclude that Ldb2a plays critical roles in controlling both negative and positive feedback on TGFβ signalling *in vivo*, discriminating the responses of the I-Smad7-mediated negative feedback from the ligand-driven positive feedback. Disruption of this apparatus makes a substantial impact on embryonic development.

### Ldb2a Provides a Feed-Forward Control to the Transcriptional Activation of *I-Smad7* by TGFβ Signals

To gain further insights into the role of Ldb2a in TGFβ signalling, we studied the regulation of *ldb2a* expression by Nodal signalling. ChIP-seq datasets show an enrichment of R-Smad3 at the *Ldb2* locus in various cell types and this enrichment can be stimulated by Activin/Nodal signalling (Fig. 6C) [8,9,48]. To study whether Ldb2a is regulated by TGFβ signals, we treated zebrafish embryos with the Nodal inhibitor SB431542, from the MBT stage. We examined expression of *ldb2a* and other Nodal targets at 0.5 and 3 hours after treatment, and showed that their expression was decreased by the blockade of Nodal signalling (Fig. 6D). Thus, an Ldb2a-mediated coherent feed-forward loop delays the activation of *Smad7* expression and the suppression of ligand gene expression. As a consequence, Ldb2a discriminates the response speed of the positive and negative feedback circuits during signal propagation, allowing the accumulation of signalling through unrestricted positive feedback before negative feedback becomes fully established.

Altogether our data suggest the following model: during the initiation and propagation of TGFβ signalling, expression of ligands is immediately up-regulated, whereas *I-Smad7* transcription is delayed by its requirement for Ldb2a, which gradually accumulates in response to the same signal (Fig. 6E). This mechanism allows signalling to self-amplify until adequate levels of Ldb2a enable the fully active Smad7-driven negative feedback, together with the direct restriction of positive feedback, to dampen excess signalling. Thus, the coherent feed-forward loop involving Ldb2a serves to delay the activation of negative feedback and the suppression of positive feedback. Despite the maternal expression of Ldb2a, this mechanism is likely to be specifically active during zygotic transcription, as phenotypes shown here were mainly caused by a splice MO that only knocks down zygotic Ldb2a. In agreement with this hypothesis, the level of maternal *ldb2a* RNA drops around the MBT stage, just before its zygotic expression increases (S2C Fig.).

We conclude that Ldb2a plays critical roles in stabilising TF complexes that control both negative and positive feedback on TGFβ signalling *in vivo*. It utilises a feed-forward circuit that discriminates the responses of the Smad7-mediated negative feedback from ligand-driven positive feedback. Disruption of this apparatus makes a substantial impact on embryonic development.

## Discussion

We have compared published ChIP-seq datasets of R-Smads and Ldb1 complex components, and shown that they co-occupy a significant proportion of the genome in different cell types, which suggests potential roles for Ldbs in TGFβ signalling. This was validated by *in vivo* studies showing that Ldb2a does indeed modulate R-Smad/TGFβ family signalling during zebrafish development.

Ldbs are non-DNA binding adaptor proteins, mediating the formation of TF complexes containing partners that are also crucial for TGFβ pathways. For example, LMO4, another non-DNA binding protein in Ldb complexes, interacts with R-SMAD1, 2, 5, 8, and Co-Smad4, in response to TGFβ signalling in human epithelial cells [27]. GATA1, a TF in Ldb complexes, has been shown to assemble with SMAD1 on BMP response elements (BREs) in human HepG2 (liver hepatocellular) cells and is required for strong activation of a BRE in the first intron of *Smad7* [26]. In addition, another TF in Ldb1 complexes, Gata2 [21], also co-occupies genomic sites with Smad1 in murine erythroid progenitors [9]. Gata1 was also shown to direct Smad1 binding to erythroid-specific genes during erythroid differentiation. Altogether these observations suggest that Ldbs may nucleate R-Smad complexes to modulate TGFβ family signalling.

Known DNA binding motifs for the Smad and Ldb complexes were found in the *Smad7* locus, including GATA, Ets, SBE, and Ebox, some of which having already been identified as active regulatory elements and required for TGFβ inducibility of I-Smads in human cells [14,26,49]. We have also shown that Ldb2a co-binds the conserved R-Smad binding site in the *I-Smad7* promoter and directly activates *I-Smad7* expression. On the other hand, Ldb2a also binds the Nodal ligand gene, *Sqt*, but represses its expression. This effect is also likely to be direct, because the expression of Nodal ligands increased immediately after the MBT when the *ldb2a* splice MO could only just have begun to have an effect. It has been shown that the first intron of *Sqt*, the promoter/proximal upstream region, and a distal upstream sequence together drive expression of the reporter gene in axial mesoderm, which does not reflect endogenous sqt expression [47], suggesting the existence of an element responsible for repressing sqt expression beyond the genomic regions used. Our ChIP-qPCR analyses showed that Ldb2a binds the *Sqt* locus, and expression of Nodal ligands/targets in the axial mesoderm was indeed increased by *ldb2a* knockdown. Thus, our findings and evidence from the literature suggest that Ldb2a represses *sqt* expression by binding to an unknown regulatory element.

The ChIP assay of Ldb2a in zebrafish has been a great challenge because Ldb proteins do not directly bind DNA. Moreover, few antibodies work for ChIP assays in zebrafish, including the zLdb2a antibody we generated. We therefore injected *ldb2a* mRNA tagged by HA or biotin at low enough doses to not cause any morphological or phenotypic disruption. The biotin-ChIP succeeded in detecting the direct binding of Ldb2a at *I-Smad7* and *Sqt*. The ChIP assays were performed during early gastrulation when, like the injected RNA, Ldb2a is active in most cells of the embryo. Thus, our observations are likely to reflect physiological interactions.

The loss of *ldb2a* in zebrafish embryos increased the phosphorylation of R-Smads and the activity of TGFβ-responsive cis-regulatory elements, as well as the expression of TGFβ target genes. These observations suggest that Ldb2a normally restricts Nodal/BMP signal transduction and our subsequent experiments show that both an increase in ligand expression and a loss of *smad7* expression contribute to the signalling perturbation seen in *ldb2a* morphants.

Knockdown of *ldb2a* led to excessive specification of mesendoderm and derivatives during development. Chemically restricting Nodal activity rescued the ectopic mesendoderm induction caused by *ldb2a* knockdown, while *bmp4* loss-of-function rescued the extra increase in lateral mesoderm specification. Therefore, Ldb2a functions in embryonic patterning through Nodal and BMP signalling. Reflecting the elevation of both signalling pathways, the effect of *ldb2a* depletion on the ventro-lateral and posterior mesendoderm fates (e.g., blood, vasculature, pronephric, and tail mesodermal tissues) was more significant than on other mesodermal lineages (e.g., trunk somites, notochord, heart, and head mesodermal tissues), as the ventro-lateral and posterior mesendoderm is formed by exposure to a combination of Nodal and BMP morphogens during gastrulation [33–35]. We have therefore shown that disruption of the Ldb2a-controlled responses to TGFβ signals makes a substantial impact on embryonic development.

Insight into the biological significance of the discrimination among R-Smad targets by Ldb2a was provided by the discovery that the *Ldb2a* gene might itself be bound by R-Smads and transcribed in response to TGFβ family signalling. Thus, an Ldb2a-mediated coherent feed-forward loop slows down the transcriptional response of *I-Smad7*. As a consequence, Ldb2a discriminates the response speeds of the positive and negative feedback circuits during signal propagation, allowing the accumulation of signalling through positive feedback before the negative feedback is fully established. Recent publications [20,50] have provided mathematical simulations and experimental investigations suggesting that coupled positive and negative feedback circuits enable cellular systems to produce optimised responses to stimuli with respect to signal duration and amplitude. Here for the first time, we have shown that the two feedback pathways can be uncoupled.

## Materials and Methods

### Ethics Statement

All animal experiments were performed under a Home Office Licence according to the Animals Scientific Procedures Act 1986, UK, and approved by local ethics committees.

### Analysis and Genome-wide Comparison of ChIP-seq Data

The ChIP-seq datasets of each protein (Smad1, Smad3, Ldb1, Scl/Tal1, Gata2, and Gata1) were downloaded from the NCBI gene expression omnibus (GEO, http://www.ncbi.nlm.nih.gov/geo). For Smad1 (ChIP-seq in murine G1ER cells), Smad3 (murine pro-B cells), and Ldb1 (murine bone marrow cells), their mapped reads on the MM8 genome (bed format) were used for peak calling analysis using MACS (version 1.4.2), while IgG was used as the negative control. Genome-wide comparison of ChIP-seq datasets was performed as previously described [8]. Briefly, the location of Smad1/3 binding (query datasets, shown in *x*-axes) (Fig. 1A and 1B) in relation to Smad1/3- or Ldb1-enriched sites (base datasets, *y*-axes) was visualised by Java Treeview with the average reads density calculated in 100-bp bins ±2.5 kb around each Smad peak position suggested by MACS. These plots show the overlaps between Ldb1 binding regions and the enriched sites of Smad1/3 genome-wide. The location of Scl, Gata2, Smad1, Gata1, and Smad3 binding in relation to Ldb1-enriched sites was also visualised (S1 Fig.). These plots show the overlaps between Ldb1 binding sites and the enriched regions of the other five proteins genome-wide.

### Zebrafish Husbandry

Wild-type and Tg(*gata1a*:GFP)^la781^ [51] embryos and adult fish were bred and maintained as described [52].

### Morpholino Injection

MO oligonucleotides (S2 Table, GeneTools) were dissolved in Milli-Q water to 25 ng/μl and stored at room temperature. Micro-injections were performed with 1 nl of each MO injected into the yolk cell of 1–2-cell stage embryos, at concentrations shown in S2 Table.

### *GFP/HA/Avi*-*ldb2a* Plasmid Generation

To generate GFP-tagged *ldb2a* mRNA for injection, the entire *ldb2a* reading frame was first cloned into the Gateway vector pDONR™221. Full-length *ldb2a* PCR fragments were generated via superscript III one-step RT-PCR system (Invitrogen) using total RNA extracted from 24 hpf embryos, with gLdb2 FWD1 and gLdb2 REV1 primers (S1 Table). Gateway cloning technology (Invitrogen) generated an *ldb2a* entry vector in pDONR221 back bone, which was sequenced and recombined with pCSGFP2 [53] to create a full length *ldb2a-GFP* plasmid, in which the *ldb2a* gene was placed immediately upstream of the GFP coding sequence.

Untagged or HA-tagged *ldb2a* fragments were amplified from 24 hpf cDNA with Ldb2-F3/Ldb2-R4, Ldb2-F2/Ldb2-R4, or Ldb2-F2/Ldb2-R5 primer pairs (S1 Table), cloned into pGEM-T easy vectors (Promega) and sequenced. To generate HA-tagged *ldb2a* mRNA for injection, *ldb2a* fragments were excised from *ldb2a*-pGEM-T entry vectors and cloned into the pCS2+ vector. To generate Avi-tagged *ldb2a* mRNA for injection, the *Flag-ldb2a-2A* fragment was amplified from the *ldb2a*-pGEM-T entry vector with 5′-Flag-ldb2a/3′-2A-ldb2a primers (S1 Table) by 2-step PCR using Phusion DNA Polymerase (NEB). The *Flag-ldb2a-2A* fragment was then annealed with an Avi-Tev-Flag oligo (S1 Table), followed by amplification of the Avi-Tev-Flag-ldb2a-2A fragment with 5′-Avi-fusionF/3′-2A-fusionR primers (S1 Table) and cloning into the pMTB2-eGFP vector using In-Fusion HD Cloning kit (Clontech).

### In Vitro Synthesis and Micro-injection of Mrna

Capped mRNA for micro-injection was in vitro transcribed from 1 μg linearised DNA template, using the Ambion mMESSAGE mMACHINE kits, and purified by QIAGEN RNeasy Micro kit, according to manufacturers’ instructions. Murine or zebrafish *Smad7* mRNAs were synthesised from published Flag-pcDNA3-mSmad7 vectors [10] or a PCS2-zSmad7 construct [12], respectively. Synthesised mRNA was aliquoted and stored at −80°C, and injected to 1-cell stage zebrafish embryos.

### SB431542 Treatment

Wild-type and *ldb2a* morphant embryos were treated with 25 μM or 100 μM SB431542 [54] from the 8-cell stage until collection at the sphere, shield, tailbud, or somitogenesis stages. Control embryos were treated with an equal volume of DMSO added to fish water.

### Whole Mount In Situ Hybridisation

Whole mount in situ hybridisation on zebrafish embryos was carried out as described [55]. Digoxigenin (DIG) or fluorescein labelled antisense RNA probes were transcribed from linearised templates using T3, T7, or Sp6 RNA polymerases (Roche). DIG and fluorescein antibodies were detected using BM-purple (Roche) or Fast Red [56], respectively.

### Western Blot

Protein extracts were prepared according to Link and colleagues [57]. Primary antibodies were used at 1:500–1:2,000 dilutions. Antibodies used included: Phospho-Smad1/5 (Ser463/465) (41D10) (Cell Signaling number 9516); Phospho-Smad2 (Ser465/467) (Cell Signaling number 3101); Smad2/3 (N-19): sc-6032 (Santa Cruz); Smad6/7 (N-19): sc-7004 (Santa Cruz).

### Luciferase Assays

50 pg SBE-luciferase [31] or Id1-BRE2-luciferase [32] constructs were co-injected with *ldb2a* MO into the streaming yolk or the yolk-free cell of 1-cell stage zebrafish embryos. 50 pg pCMV-LacZ plasmids were co-injected to normalize injection efficiency. Gastrula stage embryos were collected and washed with PBS. 20–50 embryos were homogenised in 200 μl lysis buffer (provided in the Roche Luciferase Reporter Gene Assay kit) by aspirating through 23G syringes and incubated on ice for 10 minutes, followed by a brief centrifugation. Supernatants were separated into duplicates for each assay. 50 μl and 25 μl of the supernatant were used to measure the activity of luciferase and β-galactosidase, respectively, as described [58].

### Real-Time Quantitative PCR

Total RNA was isolated with the RNAeasy Microkit (QIAGEN). Quantitative PCR was performed with SybrGreen (Applied Biosystem). Data were collected with the ABI-PRISM 7000 or 7500 Sequence Detection system. β-actin1/2, EF1α, and GAPDH were used as internal controls. The relative abundance for each sample was computed by the comparative method (∆∆Ct). Statistical analysis was by the two-sample equal variance t-test. Error bars indicate the standard deviation. Primers are listed in S1 Table. Previously published primers as described [59].

### CRISPR-sgRNA Design and Production

The sgRNA sequence targets the sense strand near the ATG of *ldb2a*. The template DNA of sgRNA was generated by PCR with Phusion polymerase (NEB) in HF buffer with a unique oligonucleotide encoding a T7 polymerase-binding site and the sgRNA target sequence (zLdb2a-ATG sgRNA F) and a reverse oligonucleotide encoding the remainder of the sgRNA sequence (sgRNA-R). In vitro transcription was performed with 100 ng purified DNA template using the Megascript T7 kit (Ambion), and sgRNA purified by phenol chloroform extraction and isopropanol precipitation. sgRNA was stored in aliquots at −80 °C. To generate *ldb2a* mutants, 1 ng NLS-Cas9 protein and 500 pg sgRNA were injected into the cell of 1-cell stage embryos. The control group was injected with 1 ng NLS-Cas9 alone.

### High Resolution Melt Analysis

Genomic DNA was extracted by homogenizing single zebrafish embryos in 20 μl of 50 mM ​NaOH, followed by incubation at 95°C for 8 minutes (gastrula embryos, older embryos require longer incubation), cooling to 4°C, and addition of 2 μl (10%) of 1 mM ​Tris-HCl (pH = 8) to neutralize the solution [60]. A 178-bp fragment spanning the sgRNA target site was amplified from control or mutant gDNA using the LC-Green Plus (BioFire Inc), HotShot Diamond PCR Master mix (Clent Lifescience), with *ldb2a* HRMA F1/ldb2a HRMA R1 primers. Details of the qPCR followed by HRMA were described previously [61]. PCR products from HRMA were cloned into pGEM-T vectors (Promega) and 16 colonies from each embryo were sequenced with T7 and SP6 primers.

### Chromatin Immunoprecipitation Followed by Sequencing or qPCR Analyses

ChIP-seq and ChIP-qPCR of endogenous Ldb1 (using anti-Ldb1 antibody N-18, Santa Cruz) on murine Flk1+ BL-CFCs isolated from day 4 EBs was performed as described [29]. 36-bp raw reads were mapped against NCBI build 37.1 of the mouse genome with ELAND (Illumina). Uniquely mapped reads were extended to 200 bp and then transformed into the genome-wide reads density (coverage) with the ShortRead Bioconductor package [62]. The coverage from ChIP and IgG control was visualized on a mirror of the UCSC genome browser.

ChIP-qPCR analyses of Ldb2a in zebrafish gastrula embryos were performed as described [63], using two different methods for the IP: (a) inject low-level (50 pg) HA-Ldb2a mRNA that does not cause any defects on its own, and then precipitate HA-Ldb2a using HA antibody-coupled dynabeads (Anti-HA tag antibody: ChIP Grade, abcam ab9110; Dynabeads Protein A for Immunoprecipitation, Novex); (b) inject 50 pg Avi-Ldb2a (Avi: biotin acceptor peptide) together with NLS-BirA (bacterial biotin ligase), and then precipitate Biotin-Ldb2a using Streptavidin-coupled Dynabeads (Dynabeads MyOne Streptavidin T1, Invitrogen). For (b), we adapted the *in vivo* biotinylation method described previously [46] for the zebrafish system.

### Previously Published ChIP-seq Datasets Used

The following previously published datasets were used: Ldb1, Scl, and Gata2 in murine bone marrow cells [21], Ldb1 in murine day 4 EB-derived Flk1+ cells [29], Smad1 and Gata1 in murine G1ER erythroid progenitors cells [9], Smad3 in murine pro-B cells [8].

## Supporting Information

S1 Data Excel spreadsheet containing, in separate sheets, the underlying numerical values and statistical analyses for Figs. 1E, 2C, 2E, 6A, 6D, S2C, S4B, and S6O. (XLSX)

S1 Fig Ldb1 complex components, R-Smad1 and R-Smad3, co-occupy genomic sites. Genome-wide comparison of different ChIP-seq datasets shows that Ldb1, Scl, Gata2, Gata1, R-Smad1, and R-Smad3 co-occupy a subset of Ldb1 binding sites across the genome. For each Ldb1 binding site (*y*-axis), the relative locations of sites bound by Ldb1 (light green), Scl (orange), Gata2 (navy), Smad1 (sky blue), Gata1 (purple), and Smad3 (red) are displayed within a 5-kb window centred on the Ldb1 bound site. Intensity at position 0 indicates co-occupancy. ChIP-seq datasets of Ldb1, Scl and Gata2 analysed here were obtained from murine bone marrow cells, while those of Smad1/Gata1 and Smad3 were performed in murine G1ER and pro-B cells, respectively [8,9].
(TIF)

S2 Fig Zebrafish *ldb2a* expression. (A) During mid-late somitogenesis, *ldb2a* is present in the notochord (red arrows) and the PLM (black arrowheads). Embryos were co-stained with *myoD* to define the stage. (B) After somitogenesis, *ldb2a* expression becomes more specific in blood vessels (red arrowhead). Maternal/zygotic *ldb2a* is ubiquitously expressed in cleavage- and blastula-stage embryos, shown by RT-qPCR analysis (C) and whole-mount in situ hybridisation (D–F). RT-qPCR primers are separated by the exon-exon boundary on the 3′ end, to reduce the genomic background.
(TIF)

S3 Fig The *ldb2a* morpholinos cause specific morphological defects and phenotypes that can be rescued by *ldb2a* mRNA. (A) MO target sites in the *ldb2a* gene are shown in red rectangles. *ldb2a* ATG MO1 targets the ATG site of *ldb2a*, whereas *ldb2a* splice MO2 spans the intron3/exon4 boundary. (B) RT-PCR analysis showed a reduction in the correctly spliced product, together with the formation of two aberrantly spliced products in *ldb2a* splice MO2 injected embryos. (C) Based on the sequences of three spliced products in *ldb2a* splice morphants, we drew the genomic structures of full length and truncated *ldb2a* with early stop codons (black asterisks). (D–E) To test the efficiency of *ldb2a* ATG MO1, it was injected with GFP-tagged *ldb2a* mRNA. The GFP fluorescence was significantly reduced in morphants. (F–K) Both *ldb2a* ATG MO and splice MO exert the same effects on expression of gastrula germ layer genes, such as *cyc* and *gata2*. (L–Q) At the shield stage, increased *cyc* expression in morphants can be rescued by co-injection of *ldb2a* mRNA. Embryonic views: (F, H and J) animal pole view with dorsal to the right; (G, I, and K) dorsal view with animal pole to the top. (R–T) During somitogenesis, the increased expression of *scl* and *bmp4* in morphants can be rescued by co-injection of *ldb2a* mRNA. Embryos were co-stained with *myoD* to help define the stage. Flat-mount embryos are shown in dorsal view, anterior to the left. ∼70% of the morphants injected with *ldb2a* mRNA showed rescued morphology during gastrulation and ∼50% showed rescued morphology during somitogenesis. (U) High resolution melt analysis (HRMA) is the quantitative analysis of the melt curve of a DNA fragment following amplification by PCR. It detects differences in the melting temperature of heteroduplexes containing insertions or deletions (indel) from wild-type homoduplexes. This technique enables a simple, fast, efficient, and sensitive detection of the indels created in the F_0_ generation. HRMA of F_0_ mosaic *ldb2a* mutant zebrafish embryos is shown here. Mosaic mutants can be easily distinguished from control embryos injected with the same amount of Cas9 without the sgRNA by a change in the shape of the melt curve. (V–W) A significant proportion of mosaic F_0_ *ldb2a* mutants showed increased expression of *cyc*, phenocopying the morphants. We are in the process of generating stable mutant lines and will further characterise the phenotype and genotype. The *wildtype* control refers to uninjected embryos that are stage matched.
(TIF)

S4 Fig Expression of *bmp4* was increased at the shield stage, when BMP activity was unaffected. (A) At shield stage, the p-Smad1/5/8 level in *ldb2a* morphants stayed the same as in wild-type siblings. (B) The relative luminescence of the Id1-BRE2-luciferease reporter in *ldb2a* morphants was unchanged at the shield stage. As a positive control for the activity of Id1-BRE2-luciferease reporter, heat-shocked Tg(hsp70I:dnBmpr-GFP) embryos displayed reduced luminescence compared to heat-shocked wild-type siblings. Error bars are based on two technical replicates in one experiment that represents three independent experiments. (C-D) Expression of *bmp4* was increased during somitogenesis (black arrows). Three independent experiments were performed, with the total number of embryos analysed indicated. The *wildtype* control refers to uninjected embryos that are stage matched.
(TIF)

S5 Fig Knockdown of *ldb2a* up-regulates the mesendoderm while reducing the ectoderm. (A–B’) Expression of a neural ectodermal gene, *otx2*, was reduced in *ldb2a* morphants. (C–F’) Expression of *mixer* was increased at the shield stage and remained evident in the endoderm of 80% epiboly *ldb2a* morphants (red arrowheads). (G–J’) Expression of *eve1* and *gsc* was significantly increased. (A–B’): three independent experiments, with the total number of analysed embryos indicated in each panel; (C–D’) and (G–J’): two independent experiments; (E–F’): one experiment, complementary to (C–D’). The *wildtype* control refers to uninjected embryos that are stage matched.
(TIF)

S6 Fig Knockdown of *ldb2a* increases specification of the ventro-lateral mesoderm and derivatives. (A–N) During somitogenesis, expression of *lmo2*, *gata2*, *fli1*, *gata1*, *draculin*, *pax8*, and *lim1* was increased in *ldb2a* morphants. (O) The mRNA level of *fli1* was significantly up-regulated in *ldb2a* morphants, shown by RT-qPCR. (P–Q) The GFP intensity in *ldb2a* MO injected Tg(gata1:GFP) embryos was increased compared to uninjected siblings. Expanded expression of GFP in *ldb2a* morphants suggests an increase in the number of Gata1 positive cells. (A–B): five independent experiments with the total number of embryos analysed indicated in each panel; (C–N): two independent experiments; (O): two independent experiments, each with three technical replicates; (P–Q): ∼100 embryos of each group were examined and ∼80% of the morphants showed the phenotype (Q). The *wildtype* control refers to uninjected embryos that are stage matched.
(TIF)

S7 Fig Endothelium lineages were increased by *ldb2a* knockdown. At 24 hpf, expression of *tie1* (A–B), *dll4* (C–D), and *deltaC* (E–F) was increased in the trunk and tail of *ldb2a* morphants. Embryonic view: lateral view of trunk and tail, with posterior to the right. ISH shown in this figure was repeated four times, with numbers of analysed embryos indicated. The *wildtype* control refers to uninjected embryos that are stage matched.
(TIF)

S8 Fig Validation of the Smad6/7 antibody and efficiency of the *smad7* MO. The level of Smad7 protein was significantly decreased in shield-stage *smad7* morphants, shown by the western blot using a Smad6/7 antibody. β-actin was the loading control. The *wildtype* control refers to uninjected embryos that are stage matched.
(TIF)

S9 Fig Low-level *ldb2a* mRNA injection does not cause morphological or phenotypic defects. Embryos injected with 50pg *ldb2a* mRNA at 1-cell stage showed no obvious morphological defect (A–B), no change in *cyc* expression at the shield stage (C–D), or expression of *scl* and *bmp4* (E–J). (C–D) was observed in three independent experiments, whereas (E–J) was observed in two independent experiments. The *wildtype* control refers to uninjected embryos that are stage matched.
(TIF)

S1 Table Primers and oligos. (XLSX)

S2 Table Morpholinos. (XLSX)

## Abbreviations

BMP: bone morphogenetic protein
BRE: BMP response element
ChIP: chromatin immunoprecipitation
hpf: hours post fertilisation
Ldb: LIM domain binding protein
MBT: mid-blastula transition
MO: morpholino
qPCR: quantitative real-time PCR
RT-qPCR: reverse transcription-qPCR
R-Smad: receptor-activated Smad
TGFβ: transforming growth factor β
TF: transcription factor
