# Supplementary figures and images for: A Novel TGFβ Modulator that Uncouples R-Smad/I-Smad-Mediated Negative Feedback from R-Smad/Ligand-Driven Positive Feedback
Source: PLoS Biol. 2015 Feb 9;13(2):e1002051. doi: 10.1371/journal.pbio.1002051 (PMC4321984; doi:10.1371/journal.pbio.1002051)

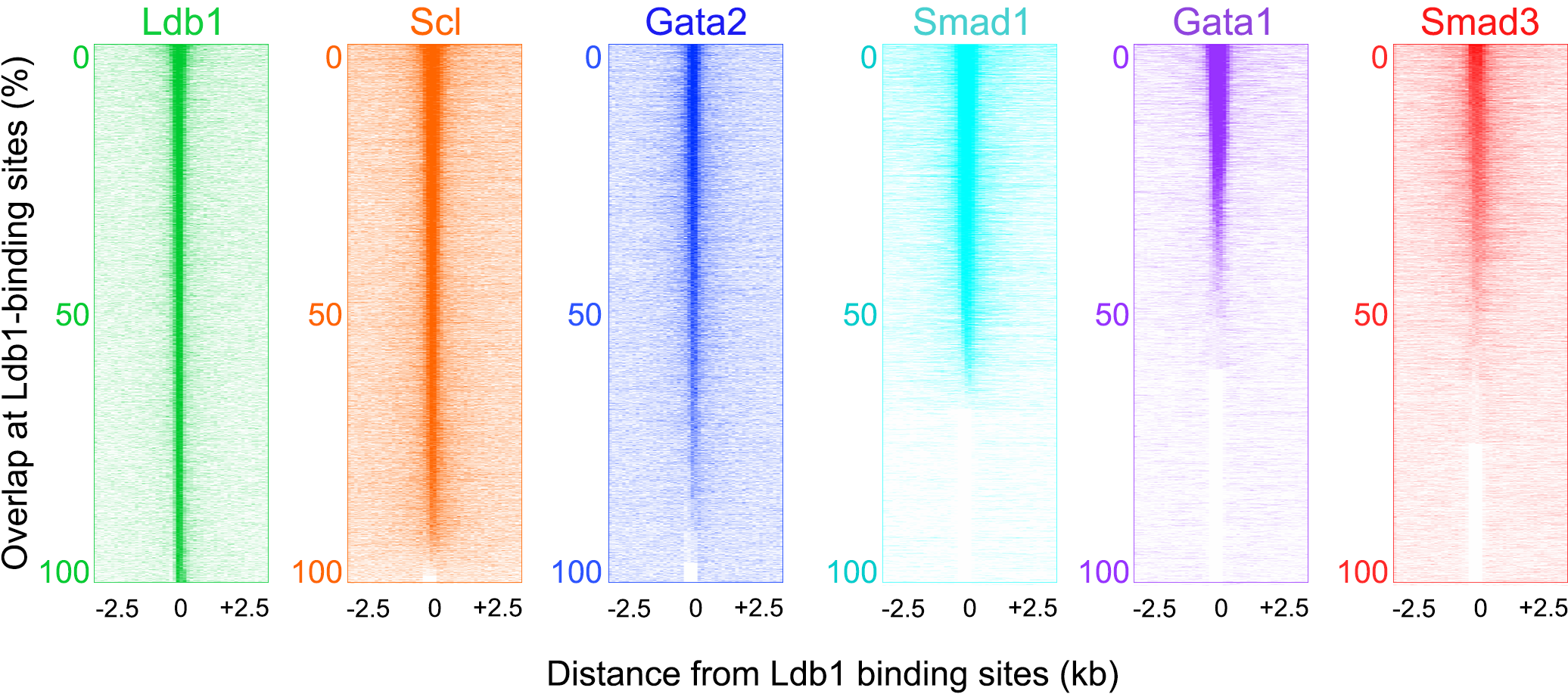

Supplement: S1 Fig — Genome-wide comparison of different ChIP-seq datasets shows that Ldb1, Scl, Gata2, Gata1, R-Smad1, and R-Smad3 co-occupy a subset of Ldb1 binding sites across the genome. For each Ldb1 binding site (y-axis), the relative locations of sites bound by Ldb1 (light green), Scl (orange), Gata2 (navy), Smad1 (sky blue), Gata1 (purple), and Smad3 (red) are displayed within a 5-kb window centred on the Ldb1 bound site. Intensity at position 0 indicates co-occupancy. ChIP-seq datasets of Ldb1, Scl and Gata2 analysed here were obtained from murine bone marrow cells, while those of Smad1/Gata1 and Smad3 were performed in murine G1ER and pro-B cells, respectively [8,9]. (TIF) [file pbio.1002051.s002.tif]

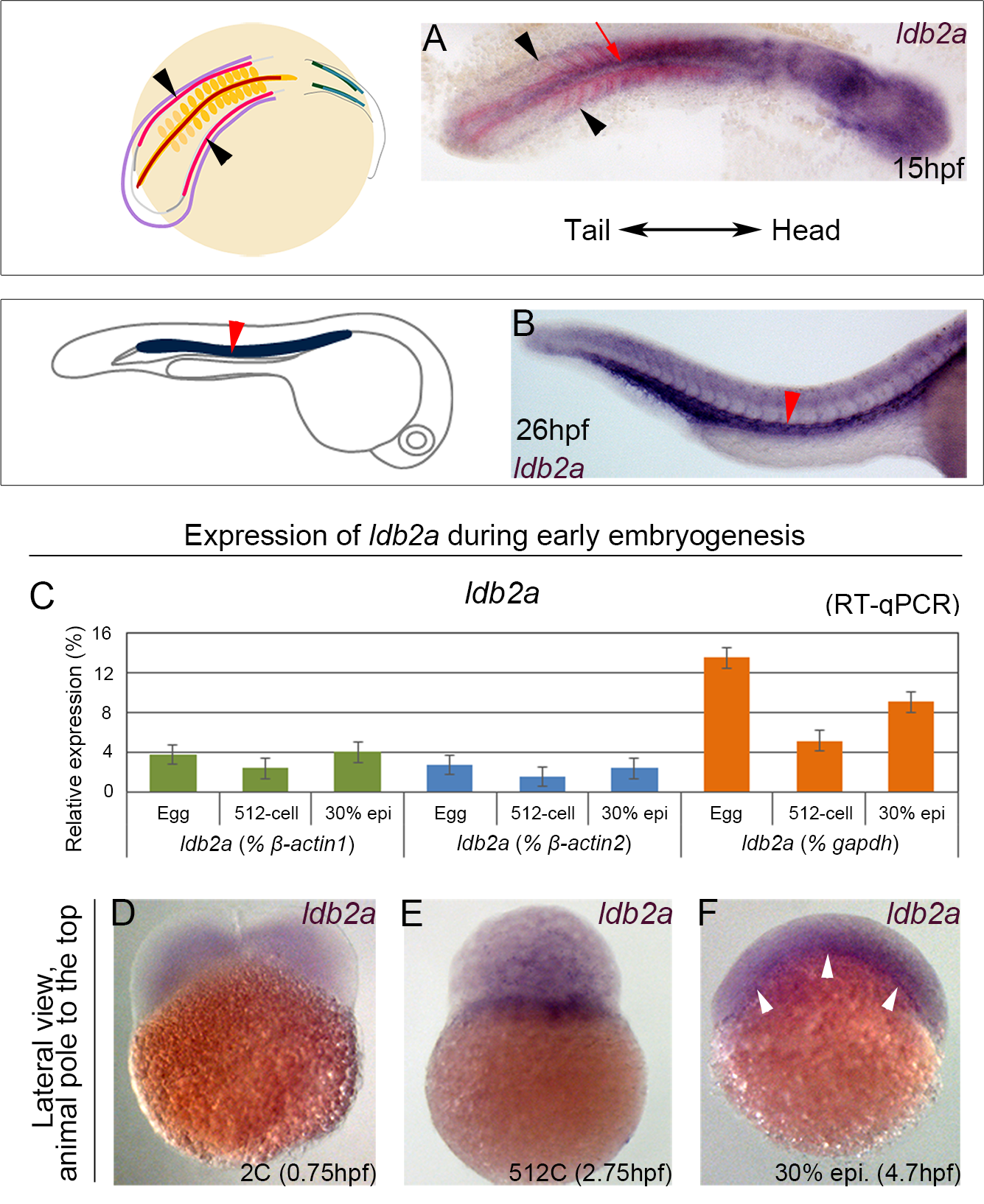

Supplement: S2 Fig — (A) During mid-late somitogenesis, ldb2a is present in the notochord (red arrows) and the PLM (black arrowheads). Embryos were co-stained with myoD to define the stage. (B) After somitogenesis, ldb2a expression becomes more specific in blood vessels (red arrowhead). Maternal/zygotic ldb2a is ubiquitously expressed in cleavage- and blastula-stage embryos, shown by RT-qPCR analysis (C) and whole-mount in situ hybridisation (D–F). RT-qPCR primers are separated by the exon-exon boundary on the 3′ end, to reduce the genomic background. (TIF) [file pbio.1002051.s003.tif]

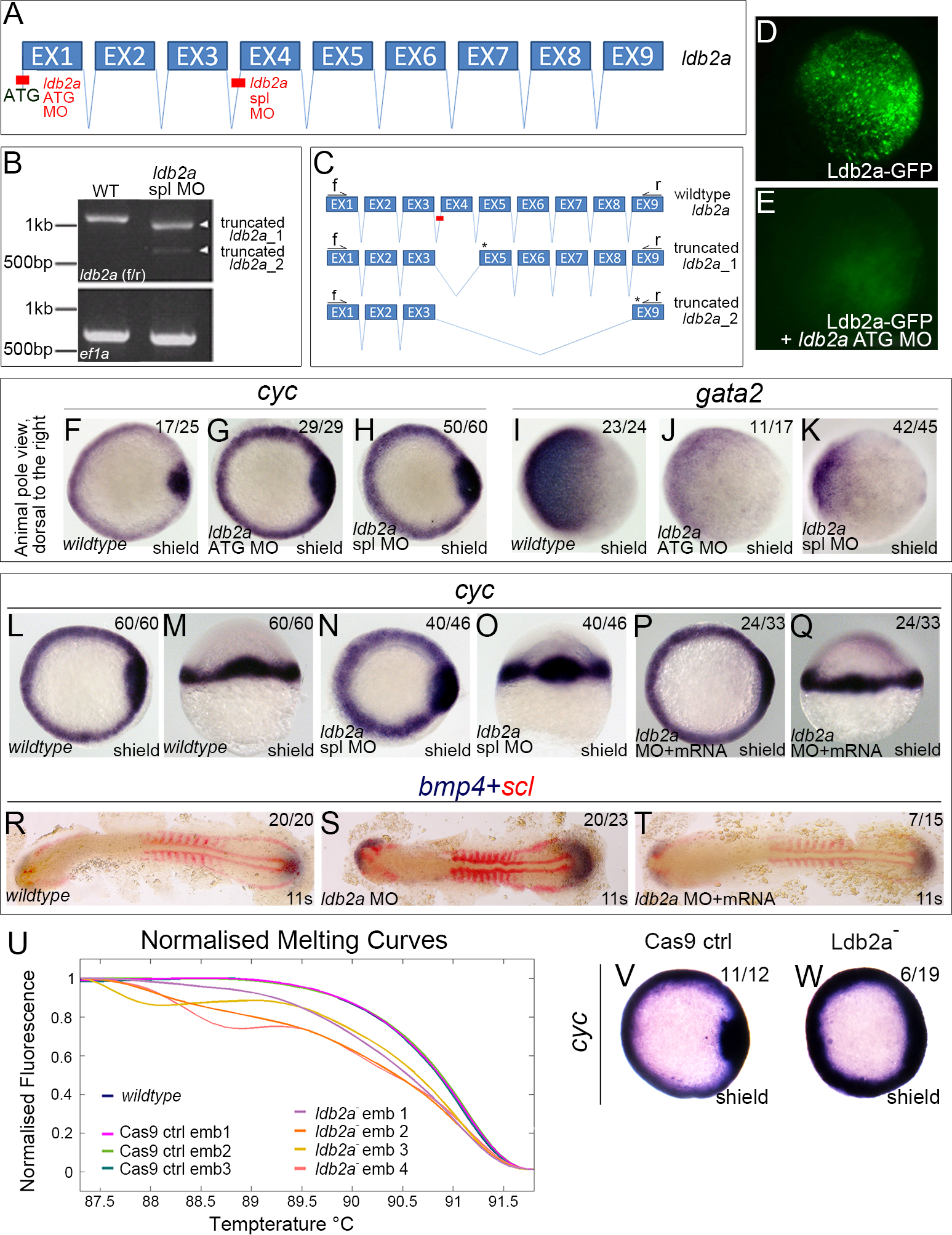

Supplement: S3 Fig — (A) MO target sites in the ldb2a gene are shown in red rectangles. ldb2a ATG MO1 targets the ATG site of ldb2a, whereas ldb2a splice MO2 spans the intron3/exon4 boundary. (B) RT-PCR analysis showed a reduction in the correctly spliced product, together with the formation of two aberrantly spliced products in ldb2a splice MO2 injected embryos. (C) Based on the sequences of three spliced products in ldb2a splice morphants, we drew the genomic structures of full length and truncated ldb2a with early stop codons (black asterisks). (D–E) To test the efficiency of ldb2a ATG MO1, it was injected with GFP-tagged ldb2a mRNA. The GFP fluorescence was significantly reduced in morphants. (F–K) Both ldb2a ATG MO and splice MO exert the same effects on expression of gastrula germ layer genes, such as cyc and gata2. (L–Q) At the shield stage, increased cyc expression in morphants can be rescued by co-injection of ldb2a mRNA. Embryonic views: (F, H and J) animal pole view with dorsal to the right; (G, I, and K) dorsal view with animal pole to the top. (R–T) During somitogenesis, the increased expression of scl and bmp4 in morphants can be rescued by co-injection of ldb2a mRNA. Embryos were co-stained with myoD to help define the stage. Flat-mount embryos are shown in dorsal view, anterior to the left. ∼70% of the morphants injected with ldb2a mRNA showed rescued morphology during gastrulation and ∼50% showed rescued morphology during somitogenesis. (U) High resolution melt analysis (HRMA) is the quantitative analysis of the melt curve of a DNA fragment following amplification by PCR. It detects differences in the melting temperature of heteroduplexes containing insertions or deletions (indel) from wild-type homoduplexes. This technique enables a simple, fast, efficient, and sensitive detection of the indels created in the F0 generation. HRMA of F0 mosaic ldb2a mutant zebrafish embryos is shown here. Mosaic mutants can be easily distinguished from control embryos injected with the s [file pbio.1002051.s004.tif]

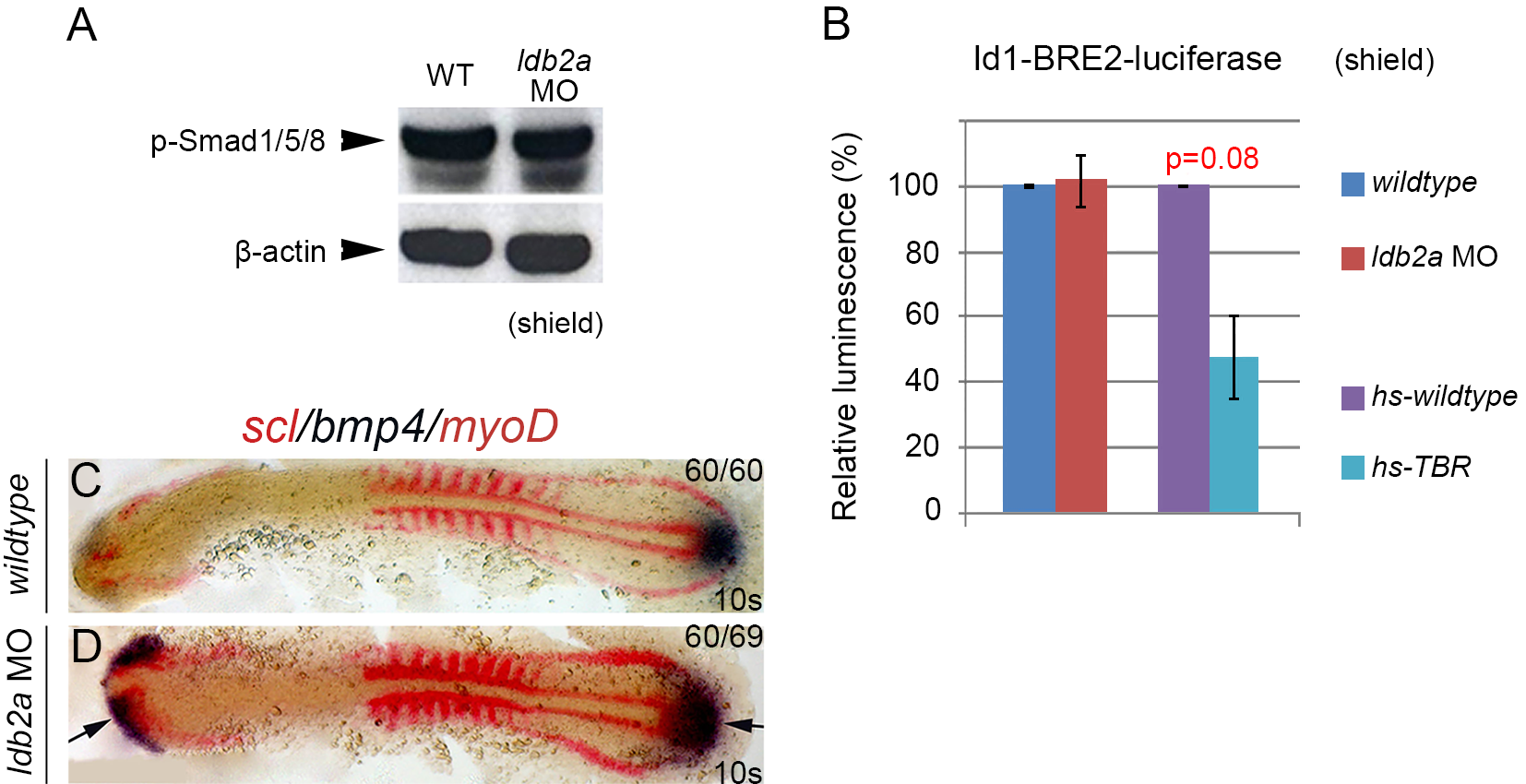

Supplement: S4 Fig — (A) At shield stage, the p-Smad1/5/8 level in ldb2a morphants stayed the same as in wild-type siblings. (B) The relative luminescence of the Id1-BRE2-luciferease reporter in ldb2a morphants was unchanged at the shield stage. As a positive control for the activity of Id1-BRE2-luciferease reporter, heat-shocked Tg(hsp70I:dnBmpr-GFP) embryos displayed reduced luminescence compared to heat-shocked wild-type siblings. Error bars are based on two technical replicates in one experiment that represents three independent experiments. (C-D) Expression of bmp4 was increased during somitogenesis (black arrows). Three independent experiments were performed, with the total number of embryos analysed indicated. The wildtype control refers to uninjected embryos that are stage matched. (TIF) [file pbio.1002051.s005.tif]

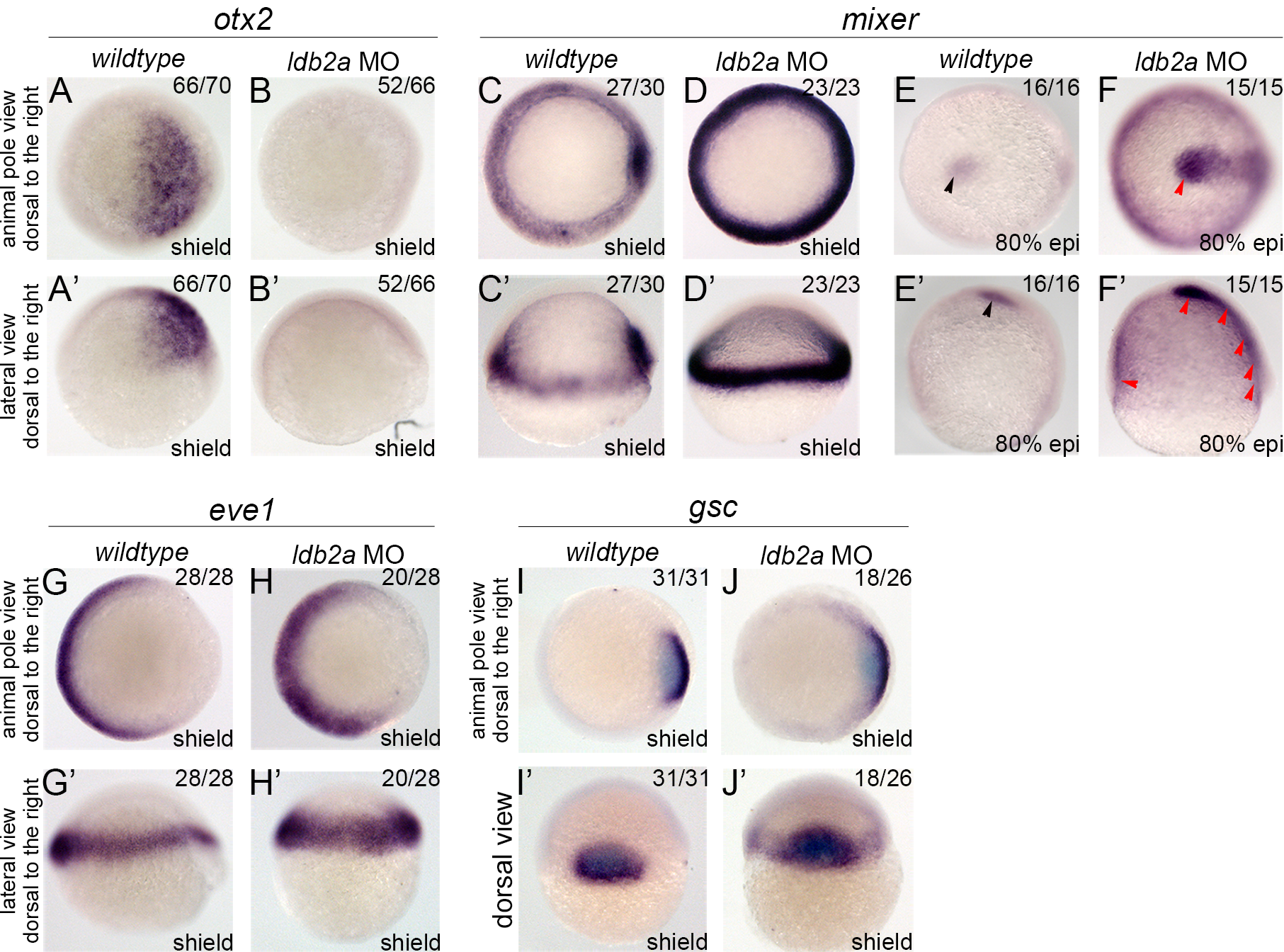

Supplement: S5 Fig — (A–B’) Expression of a neural ectodermal gene, otx2, was reduced in ldb2a morphants. (C–F’) Expression of mixer was increased at the shield stage and remained evident in the endoderm of 80% epiboly ldb2a morphants (red arrowheads). (G–J’) Expression of eve1 and gsc was significantly increased. (A–B’): three independent experiments, with the total number of analysed embryos indicated in each panel; (C–D’) and (G–J’): two independent experiments; (E–F’): one experiment, complementary to (C–D’). The wildtype control refers to uninjected embryos that are stage matched. (TIF) [file pbio.1002051.s006.tif]

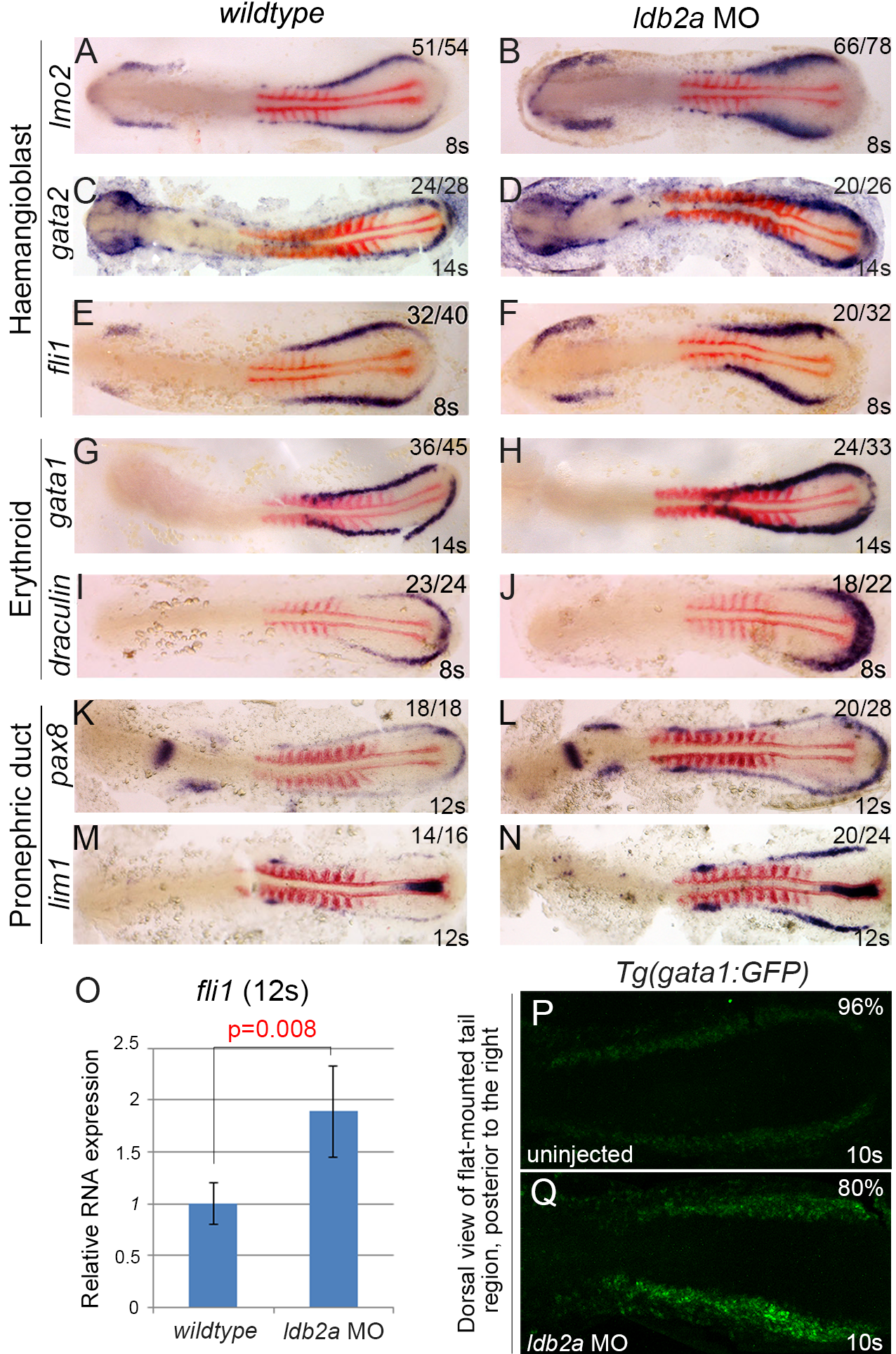

Supplement: S6 Fig — (A–N) During somitogenesis, expression of lmo2, gata2, fli1, gata1, draculin, pax8, and lim1 was increased in ldb2a morphants. (O) The mRNA level of fli1 was significantly up-regulated in ldb2a morphants, shown by RT-qPCR. (P–Q) The GFP intensity in ldb2a MO injected Tg(gata1:GFP) embryos was increased compared to uninjected siblings. Expanded expression of GFP in ldb2a morphants suggests an increase in the number of Gata1 positive cells. (A–B): five independent experiments with the total number of embryos analysed indicated in each panel; (C–N): two independent experiments; (O): two independent experiments, each with three technical replicates; (P–Q): ∼100 embryos of each group were examined and ∼80% of the morphants showed the phenotype (Q). The wildtype control refers to uninjected embryos that are stage matched. (TIF) [file pbio.1002051.s007.tif]

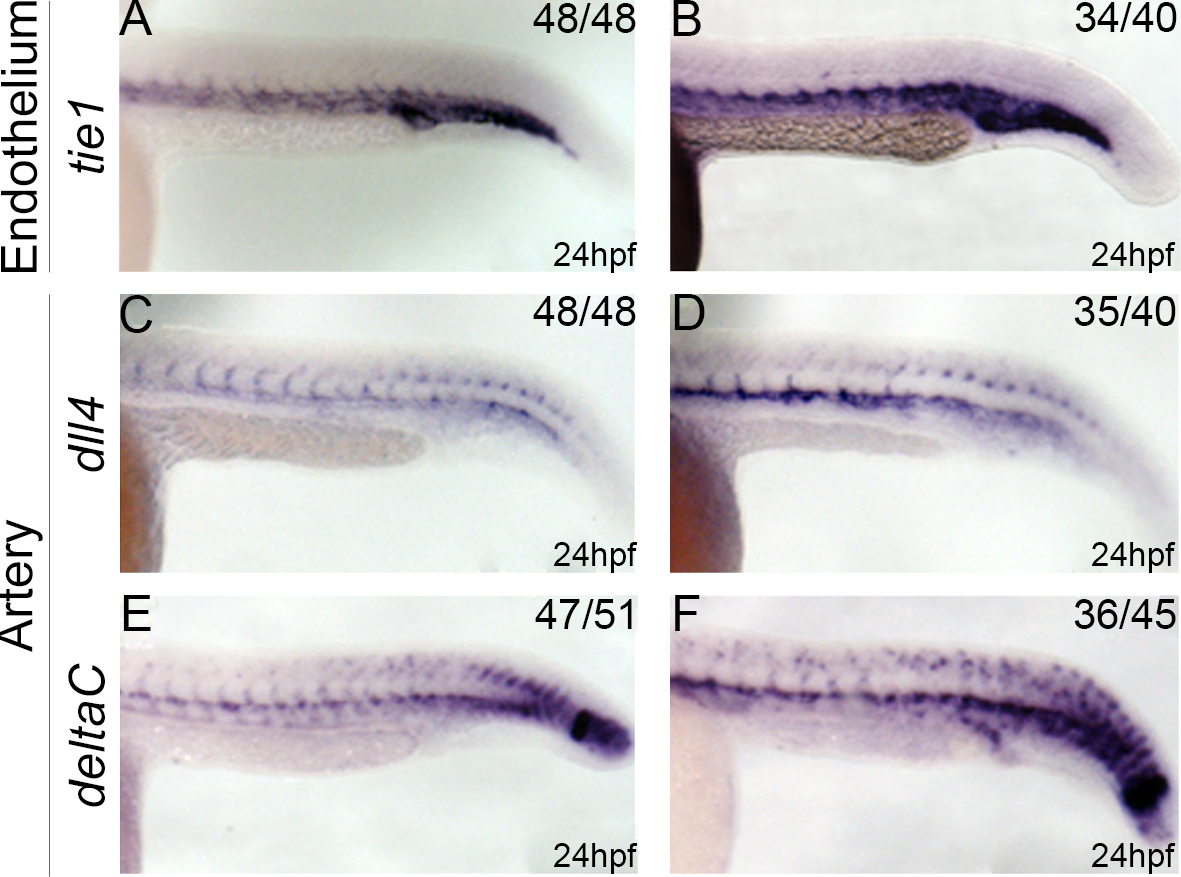

Supplement: S7 Fig — At 24 hpf, expression of tie1 (A–B), dll4 (C–D), and deltaC (E–F) was increased in the trunk and tail of ldb2a morphants. Embryonic view: lateral view of trunk and tail, with posterior to the right. ISH shown in this figure was repeated four times, with numbers of analysed embryos indicated. The wildtype control refers to uninjected embryos that are stage matched. (TIF) [file pbio.1002051.s008.tif]

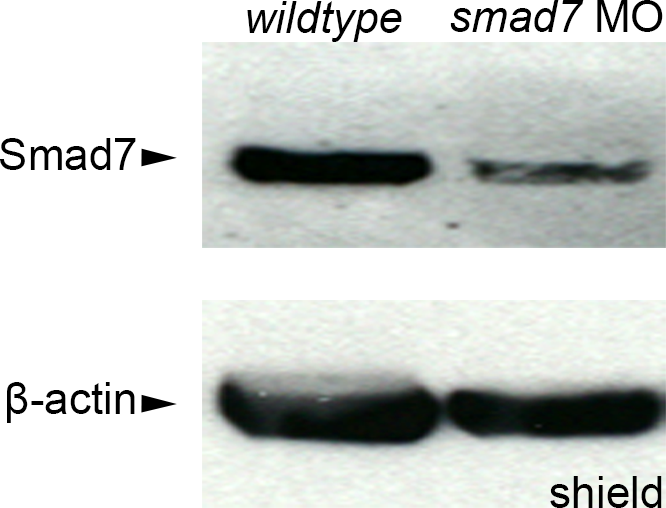

Supplement: S8 Fig — The level of Smad7 protein was significantly decreased in shield-stage smad7 morphants, shown by the western blot using a Smad6/7 antibody. β-actin was the loading control. The wildtype control refers to uninjected embryos that are stage matched. (TIF) [file pbio.1002051.s009.tif]

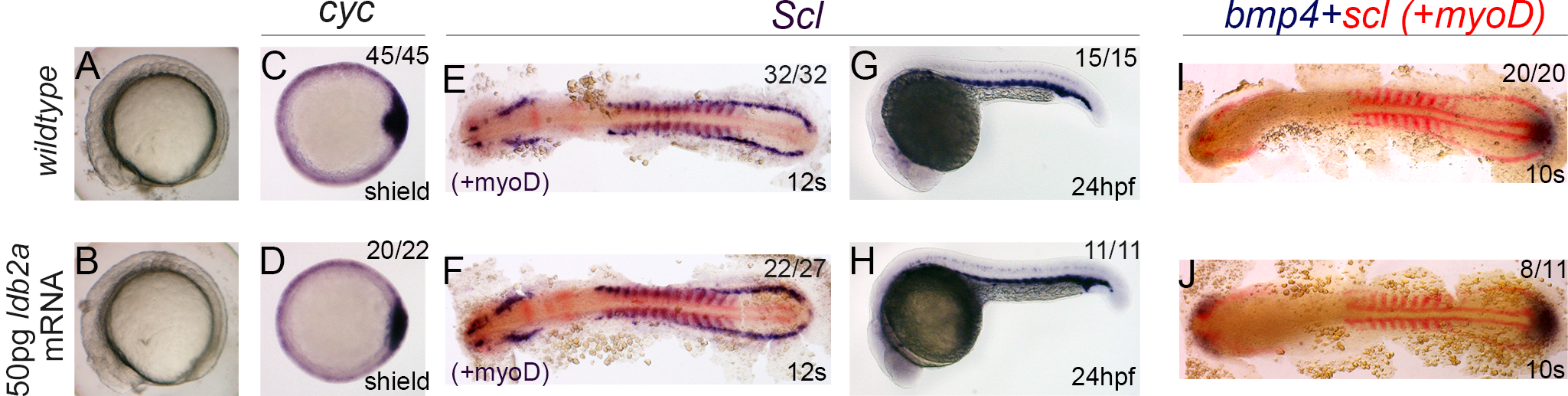

Supplement: S9 Fig — Embryos injected with 50pg ldb2a mRNA at 1-cell stage showed no obvious morphological defect (A–B), no change in cyc expression at the shield stage (C–D), or expression of scl and bmp4 (E–J). (C–D) was observed in three independent experiments, whereas (E–J) was observed in two independent experiments. The wildtype control refers to uninjected embryos that are stage matched. (TIF) [file pbio.1002051.s010.tif]
